# Involvement of MicroRNAs in Infection of Silkworm with *Bombyx mori* Cytoplasmic Polyhedrosis Virus (BmCPV)

**DOI:** 10.1371/journal.pone.0068209

**Published:** 2013-07-02

**Authors:** Ping Wu, Shaohua Han, Tao Chen, Guangxing Qin, Long Li, Xijie Guo

**Affiliations:** 1 Sericultural Research Institute, Jiangsu University of Science and Technology, Zhenjiang Jiangsu, China; 2 Quality Inspection Center for Sericulture Products, Ministry of Agriculture, Zhenjiang Jiangsu, China; Nanyang Technological University, Singapore

## Abstract

*Bombyx mori* cytoplasmic polyhedrosis virus (BmCPV) is one of the most important pathogens of silkworm. MicroRNAs (miRNAs) have been demonstrated to play key roles in regulating host-pathogen interaction. However, there are limited reports on the miRNAs expression profiles during insect pathogen challenges. In this study, four small RNA libraries from BmCPV-infected midgut of silkworm at 72 h post-inoculation and 96 h post-inoculation and their corresponding control midguts were constructed and deep sequenced. A total of 316 known miRNAs (including miRNA*) and 90 novel miRNAs were identified. Fifty-eight miRNAs displayed significant differential expression between the infected and normal midgut (P value < = 0.01 and fold change > = 2.0 or < = 0.5), among which ten differentially expressed miRNA were validated by qRT-PCR method. Further bioinformatics analysis of predicted target genes of differentially expressed miRNAs showed that the miRNA targets were involved in stimulus and immune system process in silkworm.

## Introduction

Silkworm, *Bombyx mori*, a model system for Lepidoptera, has significantly contributed to studies of insect immunology. The *B. mori* cytoplasmic polyhedrosis virus (BmCPV) is a major viral pathogen of silkworm that causes extensive damage to the sericultural industry. BmCPV belongs to the CPV subfamily, which consists of 19 distinct species (electropherotypes) within the genus Cypovirus, family Reoviridae [1]–[2]. The genome of BmCPV is composed of 10 discrete double-stranded RNA segments [3]–[4]. BmCPV infects epithelial cells of the midgut of susceptible silkworm. The infected silkworms are characterized by hypogenesis, emaciation and sluggishness. As the infection advances, white wrinkles can be observed in the posterior part of the midgut, which is the typical symptom of CPV-caused disease [5]. Until now, no effective treatment has been developed for CPV-caused disease except comprehensive prevention. The molecular mechanism of BmCPV infection is poorly understood.

MicroRNAs (miRNAs) are 18- to 25-nucleotide (nt) small noncoding RNAs that are involved in various aspects of cell and organismal biology, such as development, proliferation, apoptosis and immunity[6]–[9]. It is now well established that miRNAs are involved in host-pathogen interactions[10]–[11]. To date, over 200 virus-encoded miRNAs have been reported from various virus families [11], including insect viruses. The first insect virus encoded miRNA (HvAV-miR-1) was reported from *Heliothis virescens* ascovirus (HaAV3e) [12]. Four miRNAs has been reported from *B. mori* nucleopolyhedrovirus (BmNPV) [13]. A fifth miRNA (bmnpv-miR-5) was also predicted but its expression could not be confirmed by northern hybridization. All the BmNPV miRNAs are derived from viral ORFs encoding cathepsin, chitinase, DNA binding protein, vp80 and alkaline exonuclease [13]. In addition to virus-encoded miRNAs, host cellular miRNAs are also in relation to virus infection. The expression level of cellular miRNAs may be induced or inhibited upon virus infection and may help their replication or inhibit them through targeting viral miRNAs. A cellular miRNA (miR-32) inhibits the accumulation of primate foamy virus type 1 [14]. MiR-122 facilitates viral replication by binding to the 5′ end of the hepatitis C virus genome [15]. *Bombyx mori* miR-8 was identified as an anti-viral miRNA, which is suppressed by BmNPV following infection or transfection of a bmnpv-miR-1 mimic into the host cell [16]. *Helicoverpa zea* miR24 regulates expression of *Heliothis virescens* ascoviruses (HvAV) DNA dependent RNA polymerase II RPC2 (DdRP; ORF64) and DdRP β subunits (DdRP; ORF82) by specifically interacting with the target mRNAs [17].

The genome sequence of silkworm has been helpful in identification and characterization of specific miRNAs in *Bombyx mori*. Also, experimental studies have detected a large number of novel miRNAs that enriched the miRNA database of *Bombyx mori* [18]–[19]. However, it is not known if miRNAs are associated with BmCPV infection in silkworm. In this study, by using solexa sequencing and qRT-PCR, we revealed 316 known miRNAs and 90 novel miRNAs in midgut of the silkworm and initially screened 58 differentially expressed miRNAs between BmCPV infected and normal midguts. The results of our investigation provide insights into *Bombyx mori* miRNAs expression profiles upon BmCPV infection.

## Materials and Methods

### Silkworm Strain

Domesticated silkworm strain P50 was used in this study. They were reared at standard temperature under a photoperiod of 12 h of light and 12 h of dark up to fourth molting.

### Virus Inoculation

BmCPV viral stock was suspended in distilled water at a concentration of 10^8^ polyhedra per ml. A total of 1 ml of the viral suspension was then spread evenly on 10 pieces of mulberry leaves approximately 15 cm^2^ each in size. These leaves carrying virus were fed to 25 newly exuviated fifth instar larvae of silkworm. The dose of infection was calculated as 4×10^6^ polyhedra per larva. The control uninfected larvae were fed with the same amount of mulberry leaves with 0.9% NaC1 spread on them.

### Midgut Collection and RNA Isolation

The midguts of both BmCPV-infected and control larvae were collected at 72 h and 96 h post-inoculation by dissecting the larvae on ice. The isolated midgut was then quickly rinsed in 0.9% diethylpyrocarbonate (DEPC)-treated NaC1 to remove the attached leaf pieces before being frozen in liquid nitrogen. Total RNA was isolated from the midguts of the CPV infected larvae as well as the control uninfected larvae by using Trizol reagent (Invitrogen, USA) according to the manufacturer’s protocol. Following purification, the total RNAs were quantified by spectrophotometer and stored in −80°C.

### Small RNA Library Construction and Sequencing

Four small RNA libraries were prepared, namely 72t (BmCPV-infected midgut of silkworm at 72 h post-inoculation), 72c (control midgut of silkworm at 72 h), 96t (BmCPV-infected midgut of silkworm at 96 h post-inoculation) and 96c (control midgut of silkworm at 96 h). The small RNA library construction and deep sequencing were performed in CapitalBio Corporation (Beijing, China), using the TruSeq Small RNA Sample Preparation Kit and sequencing Kit (Illumina, CA) according to the manufacturer’s protocol. Briefly, 4 µg of total RNA was ligated with the 3′ and 5′ adapters successively. After reverse transcription, a 12 cycles of PCR reaction was performed and the products with the fragment size from 140 to 160 bp were gel-purified to enrich the 18–35 nt small RNA reverse transcription products. The resultant library was then qualified by the Agilent 2100 High Sensitivity DNA chip and quantified by Qubit and qPCR. The cluster generation and sequencing were performed on the cBot and GA*_IIx_* respectively, following the manufacturer’s standard cBot and sequencing protocols.

### Data Analysis

The adapter sequences were removed from both ends of Solexa reads. A per script was developed to remove the low quality reads, empty tags (adaptor sequence only) and tags less than 18 nt or more than 31 nt. The remaining unique reads were mapped onto the silkworm genome of SilkDB (http://silkworm.genomics.org.cn/) using the program Bowtie [20]. The perfectly matched reads were mapped onto the silkworm microRNA precursor of Sanger miRBase (Release 18) [21] by Bowtie. Two criteria were used to define the known miRNA: 1) A unique sequence must be perfectly mapped onto the precursor. 2) The start position of the alignment must be between +2 and −2 nt of the mature miRNA on the precursor. Then the perfectly matched reads were searched against the Metazoan mature microRNA of Sanger miRBase (Release 18) using the program Patscan [22]. Two mismatches were allowed to identify homologs of known miRNAs, which were defined as conserved miRNAs.

### Novel miRNA Prediction

To identify novel miRNAs, the non-coding RNA (rRNA, tRNA and snoRNA, etc), multi-mapped reads (>20) and low abundance reads (counts <2) were removed. Also, some miRNAs and minor miR* are generated from the same precursor. To identify those, we searched against the genome and combined the candidate unique reads with distances more than 150 bp in the genome. This approach was used to avoid repeated prediction. The joint genomic fragments were defined as “blocks”. For each block, the upstream and downstream150 nt of sequence were extracted for the secondary structure analysis. The Einverted of Emboss [23] was used to find the inverted repeats (stem loops or hairpin structure), with the parameters threshold = 45, match score = 3, mismatch score = 3, gap penalty = 7, and maximum repeat length = 240 as described by Jones-Rhoades and Bartel [24]. After each inverted repeat was extended 10 nt on each side, the secondary structure of the inverted repeat was predicted by the RNAfold program [25]. The unique reads in the inverted repeats were evaluated by MirCheck [24] using modified parameters ($MAX_STAR_UNPAIR = 8; $MAX_SIZEDIFFERENCE = 3; $MAX_MIR_GAP = 4; $MAX_STAR_GAP = 4).

### Targets Gene Prediction

The miRNA target genes were predicted using the approach as described by John et al [26]. A total of 14622 silkworm 3′-UTR sequences are extracted from the downstream 1000 nt of silkworm mRNA CDS (ftp://silkdb.org/pub/current/Genome/silkworm_genome_v2.0.fa.tar.gz). MiRNA candidates were used to query 3′-UTR sequences for potential target sites using miRanda program [26] with the default parameters. An energy threshold −20 kcal/mol was used to screen the high potential target candidates.

### Differentially Expressed miRNAs

The frequency of miRNA was normalized to TPM (number of transcripts per million clean tags) in order to compensate for variable numbers of tags generated for each sample. MiRNA with a P value < = 0.05 and fold change > = 1.5 or fold change < = 0.667 were considered as significantly different between 72t vs 72c and 96t vs 96c by Chi squared test.

### Quantitative Real-time PCR Analysis

Thirteen TaqMan miRNA assays were designed and ordered from Applied Biosystems. Each TaqMan miRNA assay includes specific stem-loop RT primer, forward and reverse primers and TaqMan probe (P/N: 4440886, 4427975 and 4398987). MiRNA gene specific primers were designed following the method described by Chen et al [27]. First, a miRNA-specific stem-loop RT primer is hybridized to the miRNA and the reverse transcript reaction was performed using the TaqMan microRNA RT kit (P/N: 4366596, Applied Biosystems) according to the manufacturer’s protocol. Next, Reverse transcript products were used as template for Real-time PCR using TaqMan Universal Master Mix II, no UNG kit(P/N: 4440040, Applied Biosystems). PCR reactions were run in triplicates with 2 biological replicates on ABI 7300 machine (Applied Biosystems, USA) with thermal cycling parameters at 95°C for 10 min followed by 40 cycles of 95°C for 30 s, 60°C for 1 min according to the manufacturer’s protocol. The U6 snRNA of silkworm was selected as a reference gene for normalization. A relative quantitative method (△△Ct) was used to evaluate relative expression differences.

## Results

### Global Statistics of 4 Sequenced Small RNA Libraries

**Figure 1:**
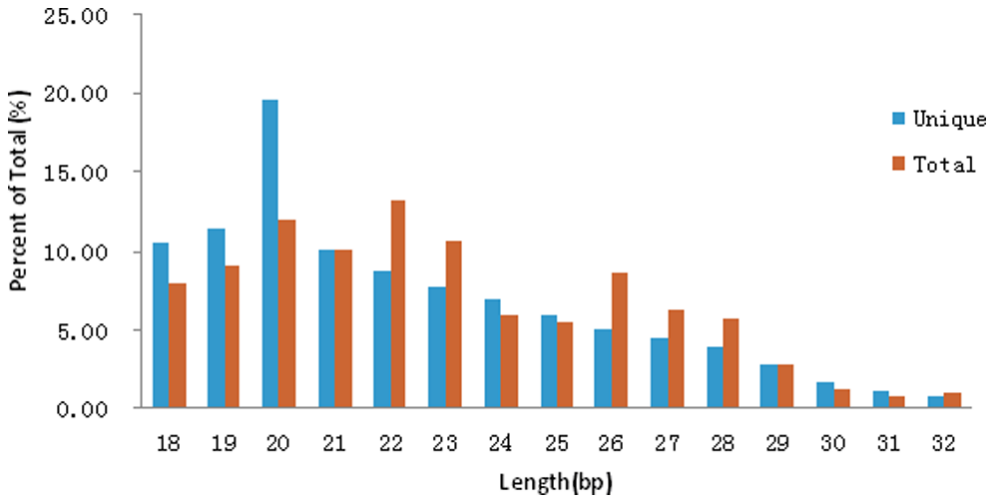
Size distribution of midgut small RNA in the four small RNA libraries.

Four small RNA libraries (72t, 72c, 96t, 96c) were sequenced and the results were shown in Table 1. After filtering the low quality reads, trimming the adaptor sequence at the 3′primer terminus and removing the 5′ adaptor contaminants formed by ligation, a total of 5383691(59.55%), 9423808(67.42%), 6215698(64.58%) and 3939590(61.96%) high-quality reads (size>18 nt) were collected (Table 1). For analysis, all identical sequence reads in each small RNA library were grouped and converted into unique reads with associated counts of the individual reads. All unique reads were mapped onto the silkworm genome using Bowtie program. The total number of unique reads from the four small RNA libraries is 2429925, comprising 1564457(64.38%) perfect matched genome reads. Size distributions based on both total abundances and unique reads were assessed and showed in Figure 1. For total abundance, more than 50% small RNAs were 18–23 bp in length. The 20-, 22-, 23-bp RNAs represented the major length classes. However, the 20 bp reads were prevailing in all reads.

**Table 1:** Expression profile of sequenced reads in four libraries.

Class	Total raw Reads	High-quality Reads	Total Unique Reads	Redundancy(%)	Perfect Matched Genome	%
72t	9040691	5383691	690998	87.16	476611	68.97
72c	13977145	9423808	1028790	89.08	701613	68.20
96t	9624507	6215698	920345	85.19	540559	58.73
96c	6358308	3939590	555537	85.90	366680	66.00
Total	39000651	24962787	2429925	90.27	1564457	64.38

### Expression Profiles of Silkworm miRNAs in Midgut

We searched known silkworm miRNAs for the four small RNA libraries by using miRBase release 18.0, which includes 562 mature silkworm miRNAs and 392 silkworm miRNA families. A total of 316 known mature miRNAs (containing miRNA*) and 201 silkworm miRNA families were identified. The results were shown in Table S1. The summary information of silkworm miRNAs in 4 sequenced small RNA libraries were shown in Table 2. We listed the known miRNA (containing miRNA family) whose total counts were >10^4^ in Table S2. The top 10 abundant miRNA occupied more than 70% of all collected miRNAs. The most abundant miRNA was bmo-miR-750 in all the 4 samples, which yielded 302954 counts, followed by bmo-miR-10 (210825 counts). Further analysis suggested that conserved miRNAs such as bmo-bantam, bmo-let-7, bmo-miR-31, bmo-miR-8 were in general highly abundant. Members of MIR-263, MIR-281 and MIR-9 were also highly expressed in the 4 samples. Using the program Patscan [22], we searched for other perfectly matched reads which had no significant match to the silkworm microRNA precursors in miRBase (Release 18.0) against the Metazoan mature microRNA of Sanger miRBase (Release 18.0). As a result, 5 conserved miRNAs, miR-2478, miR-981, miR-3351, miR-1692, miR-2774 were identified (Table S3). A total of 8 hairpin structures were confirmed including 3 of miR-3351, 2 of miR-1692 and 1 of miR-2478, miR-981 and miR-2774 respectively (Figure S1). Also, a total of 90 novel miRNAs and 98 novel hairpins were predicted by using the RNAfold program and Mircheck program (Table S4). We identified a total of 61, 62, 61, 61 novel miRNAs and 26, 27, 27, 25 novel miRNA* in 72t, 72c, 96t, 96c libraries respectively (Table 2). The frequency of most miRNAs* were significantly lower than that of relevant miRNAs because the miRNA* strand is probably degraded rapidly on exclusion from the RNA-induced silencing complex (RISC)[28]–[29]. However, the expressed levels of miRNA*, such as bmo-miR-281*, bmo-miR-2766*, bmo-miR-316*, bmo-miR-71*, bmo-miR-965*, bmo-miR-9936*, bmo-miR-9c* and bmo-miR-2808*, were either overrepresented or in similar counts relative to their relevant miRNA counts (Table S1). The abnormal expression rates of miRNA and miRNA* primarily result from similar 5′ end stability that leads to equal incorporation of either strand into the RISC and protection from degradation [30].

**Table 2:** Summary of silkworm miRNAs in 4 sequenced small RNA libraries.

	known		conserved			novel		
sample	miRNA	miRNA*	miRNA	miRNA*	hairpin	miRNA	miRNA*	hairpin
72t	178	66	5	–	8	61	26	94
72c	201	76	5	–	8	62	27	98
96t	185	72	5	–	8	61	27	97
96c	180	69	5	–	8	61	25	93

Some miRNAs were detected in only one or two small RNA libraries. For example, bmo-miR-2772b, bmo-miR-2773e, bmo-miR-932 were identified in the 72c and 96t small RNA libraries specially, but not in the 72t and 96c small RNA libraries (Table S1). This could be due to low levels of expression of these miRNAs.

### Validation of Novel Silkworm miRNAs

**Figure 2:**
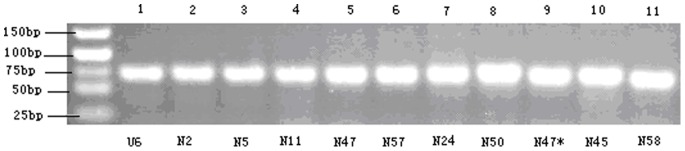
Stem-loop RT-PCR for novel miRNAs. U6: the positive control.

To validate the predicted new miRNAs, stem-loop RT-PCR assays were performed to examine whether the miRNAs were expressed in the silkworm. Ten miRNAs were selected to confirm the analysis. Primers used in this experiment are listed in Table S5. The PCR products were about 60–80 bp in length, and all the 10 miRNAs were found to be expressed in the silkworm samples (Figure 2).

### BmCPV-infection Associated miRNAs

**Figure 3:**
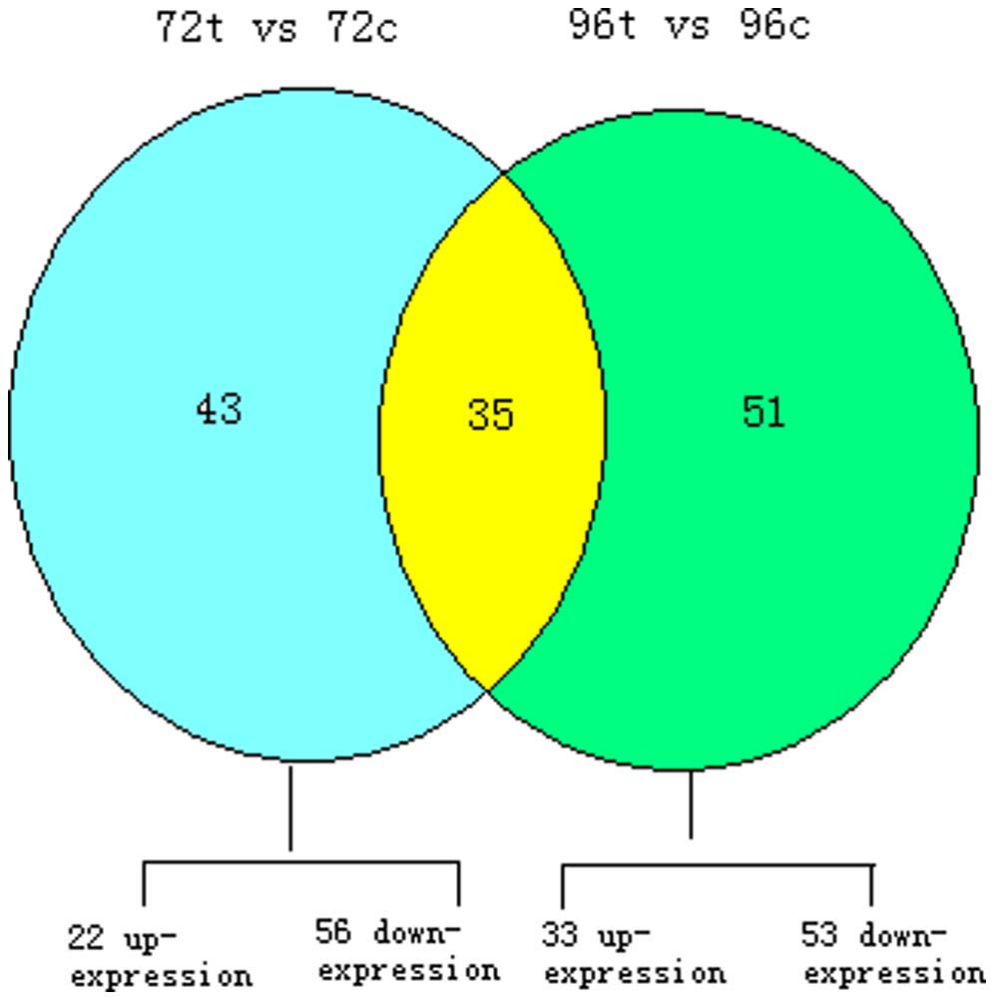
Differentially expressed miRNAs in 4 samples. 72t vs 72c: BmCPV-infected 72 h midgut against 72 h control midgut, 96t vs 96c: BmCPV-infected 96 h midgut against 96 h control midgut.

The frequency of miRNA was normalized to TPM (number of transcripts per million clean tags). Using fold change > = 1.5 or fold change < = 0.667 combined with a P value < = 0.05 as criteria, we obtained 78 differentially expressed miRNAs in BmCPV-infected 72 h midgut against control midgut (72t vs 72c), including 22 up-regulated and 56 down-regulated ones (Table S6). Total 86 differentially expressed miRNAs in BmCPV-infected 96 h midgut against control midgut (96t vs 96c) were detected including 33 up-regulated and 53 down-regulated ones (Table S7). From further analysis, we found 35 differentially expressed miRNAs both in 72t vs 72c and 96t vs 96c (Figure 3).

A total of fifty-eight differentially expressed miRNAs were obtained based on P value < = 0.01 and fold change > = 2.0 or fold change < = 0.5(Table 3, 4). Also, specific miRNA* and the corresponding miRNAs showed identical expression patterns during BmCPV-infection. For example, bmo-miR-745 and bmo-miR-745* were both down-regulated (2.8 fold) in 96t compared to 96c. Novel-58 and Novel 58* were both up-regulated (2.2 fold) in 96t compared to 96c.

**Table 3:** Differentially expressed miRNAs between BmCPV-infected 72 h midgut and normal midgut of silkworm.

MiRNA	Sequence	Length	Transcripts per million		72t vs 72c		
			72t	72c	P-Value	Ratio	Mark
bmo-miR-2843-2*	TTCGTGATCAAGCCTGACCCCTTAAT	26	2.12	0.14	0.000536	15.25	Up
bmo-miR-3203	ATGTCAGCTCAGTCAGTACACG	22	1.65	0.14	0.003177	11.86	Up
bmo-miR-3001	TAAGTTGAAAGAATTGTAGATTTTGA	26	24.54	51.94	0	0.47	Down
bmo-miR-2843	TCTAAGGAAATTAGGTCGGATACA	24	5.90	12.95	0.000343	0.46	Down
bmo-miR-307	TCACAACCTCCTTGAGTGAG	20	5.43	11.98	0.00054	0.45	Down
bmo-miR-2756	ACCCTGTAGCTGCCAAGGGGCG	22	10.62	28.69	0	0.37	Down
bmo-miR-274	GTTTGTGACCGTCACTAACGGGCAGT	26	4.48	13.51	0.000004	0.33	Down
bmo-miR-2778d*	CAGAGTACGCAAAAAACAATT	21	1.65	5.01	0.004688	0.33	Down
bmo-miR-9a	TCTTTGGTTATCTAGCTGTATGA	23	26.19	107.50	0	0.24	Down
bmo-miR-9a*	ATAAAGCTAGGTTACCGGAGTTA	23	0.94	3.90	0.003953	0.24	Down
bmo-bantam*	CTGGTTTTCATAATGATTTGACA	23	0.71	3.20	0.006951	0.22	Down
bmo-miR-2778c*	CAGAGTACGCAAAAAATCAATT	22	0.47	2.65	0.008874	0.18	Down
bmo-miR-2826	AAAAGATCGAGGATCCGATATTG	23	0.47	3.62	0.001029	0.13	Down
bmo-miR-14	TCAGTCTTTTTCTCTCTCCTA	21	508.28	1042.14	0	0.49	Down
bmo-miR-281*	AAGAGAGCTATCCGTCGACAGT	22	5430.83	10965.99	0	0.50	Down
miR-3351	TTACGTTGTAGATGCCTATG	20	0.94	3.76	0.005271	0.25	Down
Novel-46*	CCCGGGCAACCCGCTGAAACT	21	2.83	0.42	0.000585	6.78	Up
Novel-46	GAATCAGCATGTTCTCCCT	19	72.21	10.86	0	6.65	Up
Novel-11	GTTGTTGGGAAGTTGACC	18	179.10	29.38	0	6.10	Up
Novel-33	ATTTTCAGGAAGTTCACT	18	8.02	1.95	0.000001	4.12	Up
Novel-50	CGGTGTTTCGTTCCAAGCGTGCAGA	25	656.23	207.48	0	3.16	Up
Novel-41	TGATCAACGCAAAGTCGCCA	20	5.19	2.09	0.004895	2.49	Up
Novel-11*	GTGAGGTCTTCGGACCGACA	20	39.41	16.57	0	2.38	Up
Novel-10	TTCGGAACGCGAAGAGCACC	20	117.51	58.07	0	2.02	Up
Novel-7	CGTACGAGAGGAGGCATAGT	20	5.19	10.44	0.003261	0.50	Down
Novel-47*	ATCAGCGGTGGTCTGGGGTACC	22	29.50	62.38	0	0.47	Down
Novel-53	AGGATTGTGGGTGGTTCTGCC	21	11.80	26.46	0	0.45	Down
Novel-24	TTTCTCTCGGGCGTACGTTTAC	22	2792.93	7260.44	0	0.38	Down
Novel-28	CAATTCTCATTTCGGGCGTC	20	0.94	5.15	0.000288	0.18	Down

**Table 4:** Differentially expressed miRNAs between BmCPV-infected 96 h midgut and normal midgut of silkworm.

MiRNA	Sequence	Length	Transcripts per million		96t vs 96c		
			96t	96c	P-Value	Ratio	Mark
bmo-miR-2779	ATATCCGGCTCGAAGGACCA	20	25.67	8.43	0	3.05	Up
bmo-miR-275	TCAGGTACCTGAAGTAGCGCGCG	23	54.33	25.28	0	2.15	Up
bmo-miR-9a	TCTTTGGTTATCTAGCTGTATGA	23	33.15	71.80	0	0.46	Down
bmo-miR-274	GTTTGTGACCGTCACTAACGGGCAGT	26	7.23	15.84	0.00065	0.46	Down
bmo-miR-282	ACCTAGCCTCTCCTTGGCTTTGTCTGT	27	12.21	27.64	0.000003	0.44	Down
bmo-miR-2804	TTTGCATTGTAATACACTGTTA	22	9.97	23.26	0.000011	0.43	Down
bmo-miR-2778b	CAGAGTAGGCAAAAAAACAATT	22	17.45	43.15	0	0.40	Down
bmo-miR-3001	TAAGTTGAAAGAATTGTAGATTTTGA	26	32.40	83.26	0	0.39	Down
bmo-miR-745	CAGCTGCCTAGCGAAGGGCAACG	23	16.70	46.52	0	0.36	Down
bmo-miR-745*	CGGCTCATCGTGTGGCAGTTTGCT	24	3.99	11.12	0.000436	0.36	Down
bmo-miR-iab-4-5p	ACGTATACTGAATGTATCCTGA	22	4.74	14.50	0.000019	0.33	Down
bmo-miR-2838	AATTCAGCAAACTCACGGGATAA	23	1.74	6.40	0.001615	0.27	Down
bmo-miR-1a	TGGAATGTAAAGAAGTATGGAG	22	290.34	581.15	0	0.50	Down
Novel-31*	CTATACACTACCGTTACCGGC	21	6.23	1.01	0.000667	6.16	Up
Novel-56	TTTCATTGTTTCATTACTT	19	25.67	6.07	0	4.23	Up
Novel-17	ATTCGAGAACGTCGTCTGGCG	21	7.23	2.36	0.005126	3.06	Up
Novel-21	ATGGCTTGTCGTTGCGAT	18	7.23	2.36	0.005126	3.06	Up
Novel-30*	AACGCCCTTTGAGCGAAAGGG	21	10.22	3.37	0.00094	3.03	Up
Novel-45	ACGCGAGACGCGACGTCGAAGC	22	53.33	18.88	0	2.83	Up
Novel-6*-1	CATCACGGCTCCGAAGGTCCG	21	24.67	9.44	0.000003	2.61	Up
Novel-52	ATACAGTTTCGGGCACTC	18	431.65	166.53	0	2.59	Up
Novel-2	ACCCGTTCGTCGTGGATTTAAGACG	25	165.48	67.42	0	2.45	Up
Novel-30	AGAGATCTTATGTCGATGTGGCG	23	64.05	28.65	0	2.24	Up
Novel-58*	AACTTGATCATTTAGAGGAAGT	22	131.09	60.00	0	2.18	Up
Novel-58	TACCGATTGAATGATTTAG	19	194.89	90.68	0	2.15	Up
Novel-49	CGCGTGAACAGTAGTTGCTCGC	22	45.11	21.24	0	2.12	Up
Novel-6	ATTTGGATCGCGGAGATC	18	139.31	66.75	0	2.09	Up
Novel-61	TGTTCTACTTTTCTCCCGCGGT	22	7.23	17.87	0.00005	0.40	Down
Novel-50	CGGTGTTTCGTTCCAAGCGTGCAGA	25	147.04	388.34	0	0.38	Down
Novel-50*	TTACTTACTCGGTTGGGCGGAA	22	26.67	72.81	0	0.37	Down
Novel-24	TTTCTCTCGGGCGTACGTTTAC	22	844.36	2811.72	0	0.30	Down
Novel-12	ATTCACTGTAGGTATAGATA	20	6.73	23.60	0	0.29	Down
Novel-5	ATTAGGATTTTTGTTAAACC	20	39.63	148.32	0	0.27	Down
Novel-53*	TGGCAGAAGAGCCCATCGAA	20	1.00	4.05	0.008558	0.25	Down
Novel-53	AGGATTGTGGGTGGTTCTGCC	21	10.97	48.88	0	0.22	Down

**Figure 4:**
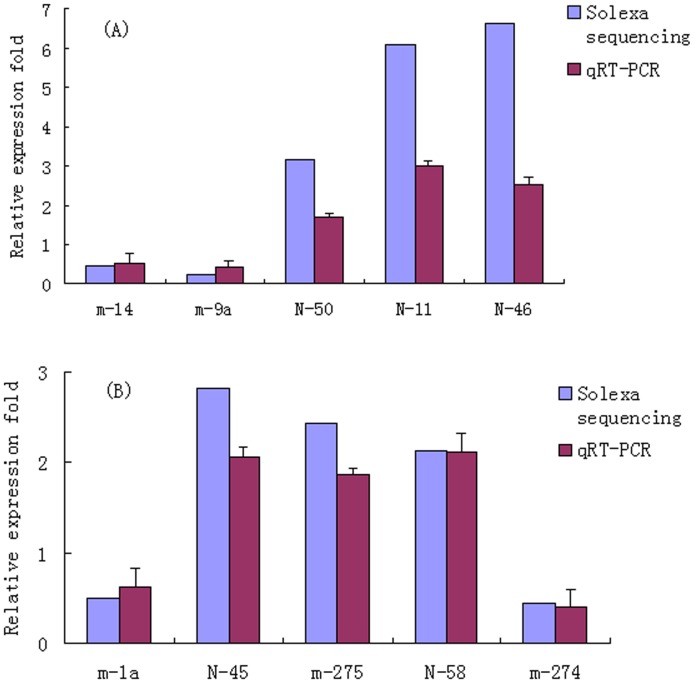
qRT-PCR results. (A) qRT-PCR confirming different expression of miRNA at BmCPV-infected 72 h, (B) qRT-PCR confirming different expression of miRNA at BmCPV-infected 96 h. Error bars represent standard deviation. qRT-PCR reactions were run in triplicates with 2 biological replicates.

To confirm the Solexa sequencing results, we selected 13 miRNAs (m-14, m-9a, m-1a, N50, N11, N46, m-282, m-274, m-745, N58, N2, N45 and U6) to perform qRT-PCR by using stem-loop RT-PCR followed by TaqMan probe to validate their differential expression. Except for m-282 and m-745, whose amplification plot were not good due to low abundance, we found that qRT-PCR results of 10 miRNAs showed the similar expressed pattern with the solexa sequencing data (Figure 4).

### Target Prediction of Differentially Expressed miRNAs

**Figure 5:**
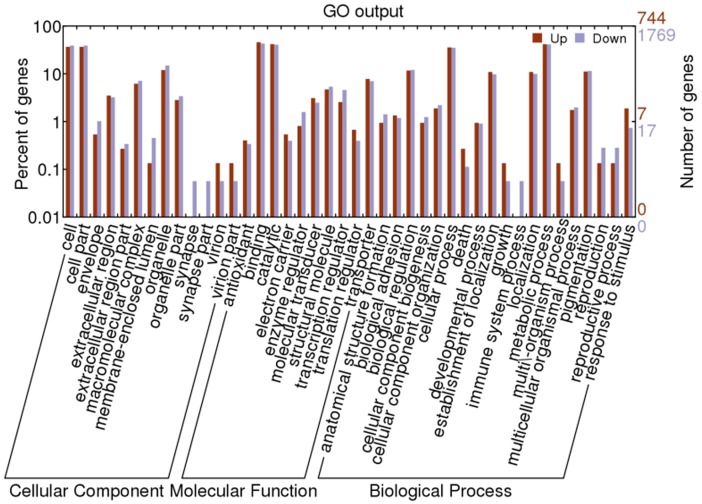
GO categories of predicted target genes of differentially expressed miRNAs at 72 h post-inoculation.

**Figure 6:**
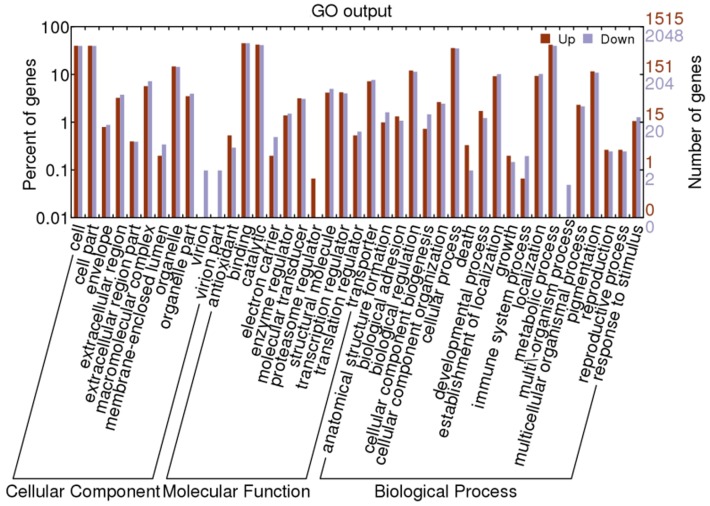
GO categories of predicted target genes of differentially expressed miRNAs at 96 h post-inoculation.

All of the differentially expressed miRNAs listed in Table 3 and Table 4 were used to search silkworm 3′-UTR sequences for predicting potential target genes using miRanda program [26]. In 72t vs 72c, a total of 744 and 1769 target genes with GO annotations were predicted from 10 up-regulated and 19 down-regulated miRNAs respectively. In 96t vs 96c, a total of 1515 and 2048 target genes with GO annotations were predicted from 16 up-regulated and 19 down-regulated miRNAs respectively. Based on gene ontology (GO) analysis, about 42 GO terms were classified (Figure 5,6). A large number of predicted target genes both in 72t vs 72c and 96t vs 96c were involved in cell, cell part, binding, catalytic, cellular process and metabolic process. However, target genes with the function of synapse and synapse part were associated only with 72t vs 72c, accounting for about 0.1% of all down-regulated miRNAs. In addition, proteasome regulator function was particularly associated with 96t vs 96c. Further analysis revealed that in 96t vs 96c, all target genes associated with virion, virion part and multi\-organism process were predicted from down-regulated miRNAs. Nevertheless, in 72t vs 72c, target genes associated with virion and virion part were predicted from both up and down-regulated miRNAs. In addition, we noticed that several up-regulated miRNAs related to immune system process were associated with miRNAs identified in 96t vs 96c.

## Discussion

Silkworm, a model system for Lepidoptera, has been studied extensively to understand innate immunity in insects [31]–[33]. However, the molecular mechanism of interactions between the host cells and BmCPV is poorly understood. Over the years, growing evidences suggest that miRNA plays a key role in host-pathogen interactions by means of regulating expression of host resistant genes or the viral genes to impair viral replication [16], [34]–[37]. Deep sequencing is an established method to study miRNAs in genome-wide manner. In this paper, we combined deep-sequencing and qRT-PCR approach to identify differentially expressed miRNAs in BmCPV-infected and uninfected midgut of silkworms.

A total of 316 known miRNAs (including miRNA*) and 90 novel miRNAs were detected by solexa sequencing in the midgut of silkworm. Among these, several conserved miRNAs such as bmo-bantam and bmo-let-7 were highly abundant suggesting that these conserved miRNAs may have important regulatory roles in *Bombyx mori.* On the other hand, about 25% of the miRNAs were least abundant (single read sequences only) suggesting that silkworm midgut has large and diverse miRNA population.

Prediction of putative target transcripts of differentially expressed miRNAs between BmCPV-infected midgut and normal midgut help us understand post-transcriptional regulation of gene expression in silkworm upon BmCPV infection. We predicted the target genes of 58 differentially expressed miRNAs whose fold change was > = 2.0 or < = 0.5. Upon BmCPV infection, a series of major physiological and pathological changes takes place in silkworm. A large number of target genes were predicted from 14622 3′-UTR sequences of silkworm genes. The predicted target genes were classified into different functional categories according to gene ontology (GO). The majority of predicted target genes were involved in binding, catalytic, cellular process and metabolic process, which are consistent with the results of microarray results of our earlier study [38]. The functional prediction of target genes between BmCPV-infected 72 h midgut and 96 h midgut revealed roles of miRNAs in susceptibility to the virus. A mature miRNA is known to regulate multiple target genes and even same gene at multiple sites[39]–[40]. We predicted that N-50 could target 6 different genes such as BGIBMGA002892-TA, BGIBMGA008315-TA, BGIBMGA009181-TA, BGIBMGA009211-TA, BGIBMGA012145-TA, and BGIBMGA013929-TA. Similarly, Bmo-miR-275 targets several genes including BGIBMGA001084-TA, BGIBMGA004198-TA, BGIBMGA009211-TA, BGIBMGA003614-TA, and BGIBMGA005392-TA. These target genes are predicted to have different functions based on gene ontologies. Also, a few miRNAs can co-regulate one gene at a time. For example, BGIBMGA005377-TA contains target sites of 3 novel miRNAs, N-24, N-30 and N-45. The results demonstrate that the regulatory role of miRNAs is complicated and probably highly networked.

Using qRT-PCR approach, 6 up-regulated miRNAs (bmo-miR-275, N-11, N-45, N-58, N-46 and N-50) and 4 down-regulated miRNAs (bmo-miR-14, bmo-miR-1a, bmo-miR-9a and bmo-miR-274) were validated for the sequence data. Analysis of putative target genes exhibits that the potential target genes to 6 differentially expressed miRNA, including bmo-miR-275, bmo-miR-14, bmo-miR-1a, N-50, N-46 and N-45, were classified into response to stimulus and immune system process based on GO analysis.

The transcript level of bmo-miR-275 in the infected midgut at 96 h post inoculation was higher than that in the control midgut. Silkworm infected with BmCPV generally exhibit stunted development. Recent studies showed that miR-275 plays important role in development and metamorphosis process of *Bombyx mori* [18], [41]. Expression pattern of Bmo-miR-275 was significantly increased in the body wall, silk glands, midgut and fat body during metamorphosis [18] and it was up-regulated from early 3rd instar to pupa but down-regulated in pupal metamorphosis of male and female [41]. In addition, miR-275 down-regulates a key differentiation factor, Bag of marbles (Bam) to ensure proper terminal differentiation in the Drosophila male germline [42]. Bryant *et al* [43] reported that miR-275 in *A. aegypti* females is regulated by a steroid hormone, 20-hydroxyecdysone and indispensable for some physiological processes including blood digestion, fluid excretion and egg development.

MiR-14 plays important roles in insect development and metamorphosis by limiting expression of its target gene, Ecdysone receptor [44]. It acts in the insulin-producing neurosecretory cell in the adult Drosophila brain to control metabolism through its direct target sugarbabe [45]. Experimental evidences showed that miR-14 plays anti-apoptotic role in Lepidoperans [46]–[47]. Apoptosis or programmed cell death is one of the strategies by which antiviral defense mechanism functions in insects [48]. Some species of Lepidopteron larvae resist baculovirus infection by selective apoptosis of the infected cells from the midgut epithelium and by sloughing off the infected cells [49]–[50]. In the present study, the expression level of bmo-miR-14 was down-regulated in the infected midgut at 72 h post inoculation suggesting that bmo-miR-14 may play a role in the interaction between silkworm and BmCPV.

Mir-9a regulates Drosophila wing development by targeting the Drosophila LIM only protein, dLMO [51]. Further study revealed that miR-9a prevents apoptosis during wing development by repressing Drosophila dLMO [52]. Recent experiment has shown that mutant flies with reduced levels of miR-9a exhibit ectopic bristles [53]. In addition, loss of miR-9a function in Drosophila peripheral nervous system leads to ectopic production of sensory organ precursors (SOPs), whereas overexpression of miR-9a results in a severe loss of SOPs [54]. In the current study, the expression of miR-9a was found to be down-regulated in BmCPV-infected 72 h midgut suggesting that miR-9a may be associated with viral infection.

The down-regulation of bmo-miR-1a and bmo-miR-274 was also detected in BmCPV-infected 96 h midgut. MiR-1 function is muscle-related as it plays central regulatory role in myoblast proliferation and differentiation in vitro[55]–[56]. Here, we found bmo-miR-1a and bmo-miR-274 may also be related to viral infection.

In general, less attention has been paid to miRNA* due to low expression and rapid degradation after processing. Interestingly, we noticed from our study that several miRNA* such as bmo-miR-281*, bmo-miR-2766*, bmo-miR-316*, bmo-miR-71*, bmo-miR-965*, bmo-miR-9936*, bmo-miR-9c* and bmo-miR-2808* were either more abundant or maintained at similar levels compared to respective mature miRNA strand. Similar results were revealed by Zhang *et al* and Liu *et al* [57]–[58]. Recently, several studies have demonstrated that miRNA* can accumulate to high levels in some cell types under certain physiological conditions and play active roles in pathogenesis of some diseases or in regulating signaling events[59]–[63]. For example, both miR171a and miR171a* are loaded onto RISC with separate regulatory outcomes [64]. The ectopic expression of miR-24-2* in MCF-7 breast cancer cells results in a suppression of cellular survival both in vivo and in vitro [65]. Therefore, more investigation are necessary to understand the biological functions of miRNA* in silkworm.

In summary, this is the first report of identification of silkworm miRNAs upon BmCPV infection. The study revealed initial information about silkworm miRNAs that are associated with BmCPV-infection and may be helpful for studying antivirus mechanisms in other Lepidoptera insects. Further knock-down experiments are necessary to elucidate underlying mechanism of BmCPV-infection regulation in the silkworm.

## Supporting Information

Figure S1 **Eight hairpin structures of 5 conserved miRNA.**
(TIF)

Table S1 **Known miRNAs in 4 small RNA libraries.** Frequency of known miRNAs of *B. mori* in each sample.
(DOC)

Table S2 **Known miRNAs with abundant expression in midgut in 4 small RNA libraries.** Known silkworm miRNAs or MiR family with total sequence counts >10^4^ in each sample.
(DOC)

Table S3 **Conserved miRNAs in 4 small RNA libraries.** Frequency of conserved miRNAs in each sample.
(DOC)

Table S4 **Novel miRNAs in 4 small RNA libraries.** Frequency of novel miRNAs in each sample.
(DOC)

Table S5 **Primers used for RT-PCR and real-time PCR.**
(DOC)

Table S6 **Differentially expressed miRNAs between BmCPV-infected 72 h midgut and normal midgut of silkworm.**
(DOC)

Table S7 **Differentially expressed miRNAs between BmCPV-infected 96 h midgut and normal midgut of silkworm.**
(DOC)
