# Supplementary figures and images for: Involvement of MicroRNAs in Infection of Silkworm with Bombyx mori Cytoplasmic Polyhedrosis Virus (BmCPV)
Source: PLoS One. 2013 Jul 2;8(7):e68209. doi: 10.1371/journal.pone.0068209 (PMC3699532; doi:10.1371/journal.pone.0068209)

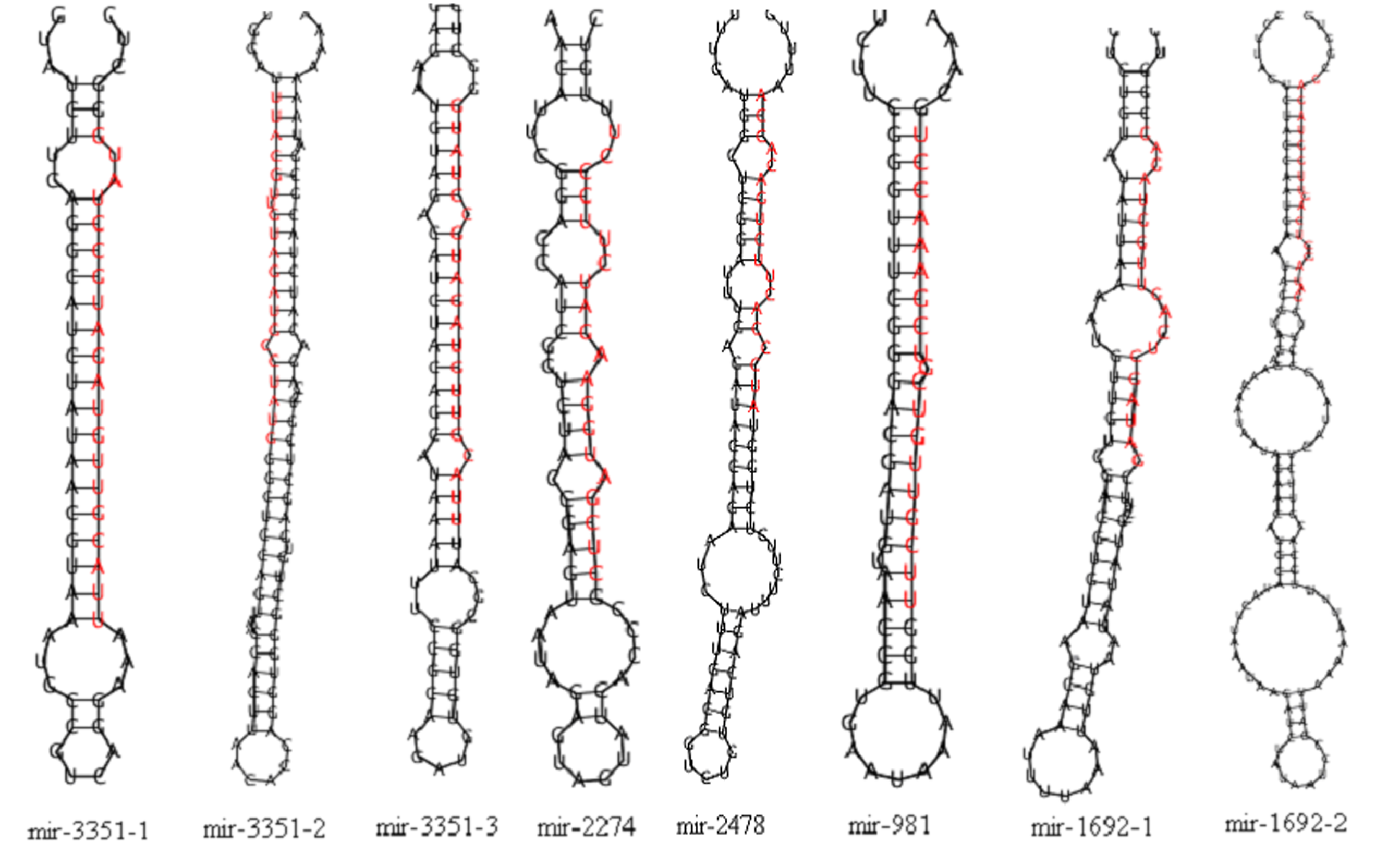

Supplement: Figure S1 — Eight hairpin structures of 5 conserved miRNA. (TIF) [file pone.0068209.s001.tif]
