# Supplementary material for: Involvement of MicroRNAs in Infection of Silkworm with Bombyx mori Cytoplasmic Polyhedrosis Virus (BmCPV)
Source: PLoS One. 2013 Jul 2;8(7):e68209. doi: 10.1371/journal.pone.0068209 (PMC3699532; doi:10.1371/journal.pone.0068209)
Supplement: Table S1 — Known miRNAs in 4 small RNA libraries. Frequency of known miRNAs of B. mori in each sample. (DOC) [file pone.0068209.s002.doc]

Table S1 Known microRNAs in 4 small RNA libraries

| MicroRNA | Sequence | Length | Normalization Counts（Transcripts per million ,TPM) | | | |
| --- | --- | --- | --- | --- | --- | --- |
|  |  |  | 72t | 72c | 96t | 96c |
| bmo-bantam | TGAGATCATTGTGAAAGCTAATT | 23 | 3333.77 | 5343.54 | 4459.29 | 5199.72 |
| bmo-bantam* | CTGGTTTTCATAATGATTTGACA | 23 | 0.71 | 3.20 | 2.24 | 5.39 |
| bmo-let-7 | TGAGGTAGTAGGTTGTATAGT | 21 | 459.67 | 620.21 | 573.46 | 695.77 |
| bmo-let-7* | CTGTATAGCCTGCTAACTTTCC | 22 | 15.34 | 20.47 | 26.42 | 27.30 |
| bmo-miR-1 | TGGGAAGTAAGGAAGCACGGAA | 22 | 0.47 | 0.42 | 0.75 | 0.00 |
| bmo-miR-10 | ACCCTGTAGATCCGAATTTGT | 21 | 9889.22 | 14757.04 | 7269.75 | 11383.76 |
| bmo-miR-10* | CAAATTCGGTTCTAGAGAGGTTT | 23 | 42.47 | 66.28 | 53.58 | 83.26 |
| bmo-miR-100 | AACCCGTAGATCCGAACTTGTG | 22 | 1954.30 | 2293.99 | 1606.97 | 1847.63 |
| bmo-miR-1000 | ATATTGTCCTGTCACAGCAGT | 21 | 0.24 | 0.14 | 0.00 | 0.00 |
| bmo-miR-11 | CATCACAGTCAGAGTTCTAGCT | 22 | 811.73 | 982.40 | 827.16 | 757.12 |
| bmo-miR-11* | TAGCACTCGACATGTGACCTGT | 22 | 9.91 | 10.30 | 11.46 | 18.54 |
| bmo-miR-1175-3p | TGAGATTCAACTCCTCCAACTTAA | 24 | 2212.21 | 2760.47 | 2950.02 | 3906.28 |
| bmo-miR-1175-5p | AAGTGGAGGTGTGATCTCTTCA | 22 | 965.35 | 987.14 | 1257.32 | 1222.65 |
| bmo-miR-12 | TGAGTATTACTTCAGGTACTGGT | 23 | 1751.13 | 1798.26 | 1894.32 | 2268.66 |
| bmo-miR-124 | TAAGGCACGCGGTGAATGCCAAG | 23 | 0.00 | 0.14 | 0.00 | 0.67 |
| bmo-miR-133 | TTGGTCCCCTTCAACCAGCTGT | 22 | 28.79 | 41.08 | 39.38 | 36.74 |
| bmo-miR-137 | TTATTGCTTGAGAATACACGTA | 22 | 1.65 | 2.23 | 1.99 | 4.72 |
| bmo-miR-13a | TATCACAGCCACTTTGATGTG | 21 | 7.08 | 3.76 | 6.23 | 6.40 |
| bmo-miR-13a* | CCTGTCAAAGCGGCGGTGAAA | 21 | 0.00 | 0.70 | 0.25 | 1.35 |
| bmo-miR-13b | TATCACAGCCATTTTTGACGAGT | 23 | 11.33 | 18.38 | 23.18 | 30.68 |
| bmo-miR-13b* | TCGTAAAAATGGCTGTGTCGTG | 22 | 0.00 | 0.42 | 0.75 | 0.00 |
| bmo-miR-14 | TCAGTCTTTTTCTCTCTCCTA | 21 | 649.86 | 1042.14 | 1205.48 | 1056.12 |
| bmo-miR-14* | CGGGGAGAGAAATCGACGAGGCT | 23 | 37.99 | 41.77 | 46.11 | 36.07 |
| bmo-miR-184 | ACTGGACGGAGAACTGATAAGGGC | 24 | 2531.00 | 3165.82 | 2173.45 | 3210.17 |
| bmo-miR-184* | CCTTGTCATTCTTCAGGCCCTG | 22 | 0.94 | 2.51 | 1.74 | 2.02 |
| bmo-miR-190 | AGATATGTTTGATATTCTTGGTT | 23 | 54.98 | 79.09 | 73.27 | 103.49 |
| bmo-miR-190* | CCCGGGAATCAAACATATTACTCT | 24 | 7.79 | 9.47 | 4.74 | 5.06 |
| bmo-miR-1a | TGGAATGTAAAGAAGTATGGAG | 22 | 420.73 | 693.74 | 324.48 | 581.15 |
| bmo-miR-1a* | CCGTGCTTCCTTACTTCCCAT | 21 | 0.47 | 0.42 | 0.50 | 0.34 |
| bmo-miR-252 | CTAAGTACTAGTGCCGCAGGAG | 22 | 2.36 | 3.20 | 2.49 | 3.71 |
| bmo-miR-263a | AATGGCACTGGAAGAATTCAC | 21 | 8841.05 | 9211.73 | 4804.46 | 5140.05 |
| bmo-miR-263a* | CGTGATCTCTTAGTGGCATCAC | 22 | 8.73 | 9.19 | 9.47 | 10.11 |
| bmo-miR-263b | CTTGGCACTGGGAGAATTCAC | 21 | 4.01 | 5.01 | 2.74 | 2.36 |
| bmo-miR-263b* | GTGAATTTCCCGATGCCTTAG | 21 | 0.24 | 0.97 | 0.75 | 0.67 |
| bmo-miR-2729 | ATTTTATGAGGTCGGTCCAT | 20 | 0.24 | 0.28 | 0.00 | 0.34 |
| bmo-miR-2733a | TCACTGGGTGCATGATGATTGT | 22 | 0.00 | 0.00 | 0.25 | 0.00 |
| bmo-miR-2733c | TCACTGGGAGAGTGATGATTGC | 22 | 0.71 | 1.25 | 1.25 | 1.35 |
| bmo-miR-2733d | TCACTGGGTGTATGAAGATTGT | 22 | 0.00 | 0.00 | 0.75 | 0.00 |
| bmo-miR-2733e | TCACTGGGAATGTAATAGCTAT | 22 | 0.00 | 0.56 | 0.75 | 0.00 |
| bmo-miR-2733f | TCACTGGGTATGTAATGACAGT | 22 | 0.00 | 0.00 | 0.00 | 0.67 |
| bmo-miR-2733h | TCACTGGGTGTATGATGATTG | 21 | 0.00 | 0.14 | 0.25 | 0.00 |
| bmo-miR-2733i | TCACTGGGAATGTAATGACTAT | 22 | 0.47 | 0.42 | 1.25 | 0.67 |
| bmo-miR-2736 | TACCAAAACGTCGATGGTACCAGCAG | 26 | 0.24 | 0.00 | 0.00 | 0.00 |
| bmo-miR-2738 | ATCTTTTAGAACGGCCATCTGATG | 24 | 4.72 | 3.90 | 7.48 | 4.38 |
| bmo-miR-2739 | GAGATGTGGATATTATGGTGTGGAGG | 26 | 0.24 | 0.14 | 0.00 | 0.00 |
| bmo-miR-274 | GTTTGTGACCGTCACTAACGGGCAGT | 26 | 4.48 | 13.51 | 7.23 | 15.84 |
| bmo-miR-274* | TCGTTTTGGCGATCGCAAAATG | 22 | 0.00 | 0.97 | 0.25 | 0.34 |
| bmo-miR-2744 | TTTGAGAGTCCTAGCTAG | 18 | 1.89 | 2.09 | 0.50 | 1.01 |
| bmo-miR-2745 | TAAATTCGGTCTTTCGGGC | 19 | 108.07 | 156.93 | 168.97 | 205.29 |
| bmo-miR-275 | TCAGGTACCTGAAGTAGCGCGCG | 23 | 29.97 | 45.81 | 54.33 | 25.28 |
| bmo-miR-275* | CGCGCTACTCCGGCGCCAGGACT | 23 | 5.43 | 7.66 | 45.61 | 26.97 |
| bmo-miR-2753 | TCAAATGGAAGAAGCTGGTA | 20 | 0.00 | 0.14 | 0.00 | 0.34 |
| bmo-miR-2754 | TCTGGTATTGACTGTAATAG | 20 | 1.89 | 2.37 | 1.50 | 0.67 |
| bmo-miR-2755 | CACCCTGTCAGACCATACTTGTT | 23 | 91.32 | 123.79 | 119.87 | 172.26 |
| bmo-miR-2755* | CAAGGTGGCCTAGCAGAGTGTT | 22 | 2.60 | 5.15 | 1.74 | 4.38 |
| bmo-miR-2756 | ACCCTGTAGCTGCCAAGGGGCG | 22 | 10.62 | 28.69 | 17.69 | 30.00 |
| bmo-miR-2756* | CCCCTCGGCTGCTACATTGTAT | 22 | 0.24 | 0.97 | 1.00 | 1.35 |
| bmo-miR-2757 | ACGGGAATGGTCACTTACAACT | 22 | 9.44 | 12.53 | 7.48 | 8.43 |
| bmo-miR-2757* | CTGAAAGTGTCCATTTCCGTT | 21 | 7.55 | 10.17 | 4.98 | 9.44 |
| bmo-miR-2758 | ACTTGGTAGAACACGTAGTAAG | 22 | 1325.20 | 1811.35 | 1617.94 | 1905.61 |
| bmo-miR-2758* | CCCTGCGTGTTCTACCAAGTTA | 22 | 22.65 | 29.10 | 32.40 | 38.09 |
| bmo-miR-2759 | TGAAAGATCATAGAATGCGAAA | 22 | 1.18 | 2.92 | 2.24 | 2.02 |
| bmo-miR-2760 | TTTTTGGTTGTTAAGTTCCATT | 22 | 23.60 | 30.36 | 34.89 | 42.14 |
| bmo-miR-2760* | CGAGGCTTAATTGAACCAAAAAGC | 24 | 4.48 | 5.71 | 3.74 | 8.09 |
| bmo-miR-2761 | TGTGTGGAACCGTCGTCGATGGA | 23 | 0.24 | 0.14 | 0.25 | 0.00 |
| bmo-miR-2761* | CATCGAGACGGTTCCATACA | 20 | 0.47 | 0.14 | 0.00 | 0.67 |
| bmo-miR-2762 | GTACGTCGGGAAATGTACGGTA | 22 | 0.24 | 0.00 | 0.00 | 0.00 |
| bmo-miR-2763 | TATTATGCTCATTTCTTTGGAT | 22 | 0.00 | 0.42 | 0.50 | 0.00 |
| bmo-miR-2765 | TGGTAACTCCACCACCGTTGGC | 22 | 2.60 | 2.09 | 0.25 | 1.35 |
| bmo-miR-2766 | TCAGTCTTGTCGAATGGTG | 19 | 986.35 | 1353.78 | 1091.58 | 1405.69 |
| bmo-miR-2766* | CCGCCCTTCGTCTTGACTGGCG | 22 | 968.89 | 1432.87 | 1596.01 | 1793.69 |
| bmo-miR-2767 | CAAGTAAATCTCGTGCGGTTTG | 22 | 0.47 | 0.42 | 0.25 | 0.34 |
| bmo-miR-2768 | ATTGGTTAAGATATTGCATCGT | 22 | 0.00 | 0.00 | 0.00 | 0.67 |
| bmo-miR-2769 | ATATATTATCAGATTTTCGGTC | 22 | 25.48 | 38.43 | 46.60 | 69.78 |
| bmo-miR-277 | TAAATGCACTATCTGGTACGACA | 23 | 70.08 | 119.75 | 118.38 | 162.48 |
| bmo-miR-277* | TCGTGCCAGGAGTGCGTTTGC | 21 | 0.71 | 0.14 | 0.75 | 2.36 |
| bmo-miR-2770 | TAGATAGAATTCGTCGGTGGATAA | 24 | 0.00 | 0.00 | 0.25 | 0.00 |
| bmo-miR-2771 | ATATTATGCAGAACACTAATGT | 22 | 0.00 | 0.00 | 0.50 | 0.00 |
| bmo-miR-2772a | TGTTTGAGTTCTGGACCTGGA | 21 | 3.07 | 2.09 | 2.24 | 2.36 |
| bmo-miR-2772b | TGTTTGAGTTCTGGATCTGGA | 21 | 0.00 | 0.42 | 1.50 | 0.00 |
| bmo-miR-2774a | CTCGGTGGAAGATCGTCCCT | 20 | 24.07 | 38.57 | 29.16 | 26.29 |
| bmo-miR-2774b | CTCGGTAGAAGATCTTCCCTTT | 22 | 0.00 | 0.14 | 0.25 | 0.00 |
| bmo-miR-2774c | CTCGGTGGAAGACCTTCCCTT | 21 | 1.42 | 2.65 | 1.50 | 1.69 |
| bmo-miR-2775a | CGCGGGAGAAAAGTAGGACATT | 22 | 1.65 | 3.34 | 3.24 | 6.40 |
| bmo-miR-2775b | AGTAGGACGTTTCCTCCCGCGTG | 23 | 0.71 | 1.25 | 0.75 | 0.34 |
| bmo-miR-2776 | AGAGTACGCAAAAAAACAATT | 21 | 1.42 | 2.51 | 3.49 | 5.39 |
| bmo-miR-2777 | AGGGACCTTGCCTGGGTTAT | 20 | 0.00 | 1.11 | 1.00 | 1.35 |
| bmo-miR-2777* | AGAGCGCAGGGGAGGTGCTTAG | 22 | 0.24 | 0.14 | 0.25 | 0.34 |
| bmo-miR-2778a | CAGAGTACGCAAAAAAACAATT | 22 | 833.91 | 1228.04 | 1141.93 | 1308.95 |
| bmo-miR-2778a* | GTTTTTTTGCATATCCTGCA | 20 | 17.70 | 29.52 | 47.85 | 52.59 |
| bmo-miR-2778a-2* | GTTTTTTTGTATATCCTGC | 19 | 0.00 | 0.14 | 0.50 | 0.00 |
| bmo-miR-2778a-4* | GTTATTTTGCATATCCTGCA | 20 | 0.47 | 0.70 | 0.25 | 1.01 |
| bmo-miR-2778b | CAGAGTAGGCAAAAAAACAATT | 22 | 20.53 | 33.00 | 17.45 | 43.15 |
| bmo-miR-2778b* | GTTTTTTTGCATATCCTGCA | 20 | 17.70 | 29.52 | 47.85 | 52.59 |
| bmo-miR-2778c | GTTTTTTTGCATATCCTGCA | 20 | 17.70 | 29.52 | 47.85 | 52.59 |
| bmo-miR-2778c* | CAGAGTACGCAAAAAATCAATT | 22 | 0.47 | 2.65 | 3.74 | 4.38 |
| bmo-miR-2778d | GTTTTTTTGCATATCCTGCA | 20 | 17.70 | 29.52 | 47.85 | 52.59 |
| bmo-miR-2778d* | CAGAGTACGCAAAAAACAATT | 21 | 1.65 | 5.01 | 3.99 | 2.36 |
| bmo-miR-2779 | ATATCCGGCTCGAAGGACCA | 20 | 14.87 | 15.04 | 25.67 | 8.43 |
| bmo-miR-278 | TCGGTGGGATCTTCGTCCGTTT | 22 | 163.76 | 218.62 | 226.79 | 212.71 |
| bmo-miR-278* | CCGGACGAACTTCCCAGCTCGG | 22 | 1053.13 | 1186.40 | 1950.15 | 1350.07 |
| bmo-miR-2780a | AAGGGTGTTAAATGAAGACTTAT | 23 | 2.60 | 2.23 | 4.24 | 2.36 |
| bmo-miR-2780a* | AATCGTTTGAATGCTCGTCTAA | 22 | 0.71 | 2.09 | 1.50 | 4.05 |
| bmo-miR-2780b | AAGGGTGTTAAATGAAGACTTAA | 23 | 2.60 | 2.09 | 4.24 | 2.36 |
| bmo-miR-2781 | TACCAGATGTTAGATTGAAGGAG | 23 | 0.47 | 0.00 | 0.25 | 0.34 |
| bmo-miR-2787 | TGAGAAAGTTCCACGATGTGCATG | 24 | 4.96 | 7.38 | 5.98 | 7.75 |
| bmo-miR-2787* | TATCTTGAATTTGATTAGCACTAAA | 25 | 0.24 | 0.14 | 0.00 | 0.34 |
| bmo-miR-2791 | TTTTCATAACAGCTCTGTCAGC | 22 | 11.33 | 15.32 | 8.97 | 13.15 |
| bmo-miR-2792-3p | TAGATTAGATAGCGATTCCATT | 22 | 0.24 | 1.81 | 0.50 | 0.67 |
| bmo-miR-2792-5p | TGGTATCGTTCTTTAATCAACA | 22 | 0.24 | 0.28 | 0.75 | 0.00 |
| bmo-miR-2794 | ACGGCGACTTTTCGTAGCGA | 20 | 0.00 | 0.00 | 0.25 | 0.00 |
| bmo-miR-2795 | CAAGTTTGGTGATACGCGGGCGC | 23 | 4.48 | 7.24 | 4.74 | 7.42 |
| bmo-miR-2797a | ATAAGTAGGTTTTATTCGACTT | 22 | 0.00 | 0.28 | 0.00 | 0.00 |
| bmo-miR-2797b | ATAAGTAGGTTCTTATTCGGCT | 22 | 0.71 | 0.84 | 0.75 | 1.01 |
| bmo-miR-2797d | ATAAGTAGACATTGTCCGGCTT | 22 | 0.24 | 0.14 | 0.50 | 1.01 |
| bmo-miR-2799 | AGAGGTTTATGAACATGATGAG | 22 | 0.00 | 0.14 | 0.00 | 0.34 |
| bmo-miR-279a | TGACTAGATCCACACTCAT | 19 | 50.03 | 82.02 | 66.54 | 119.33 |
| bmo-miR-279b | TGACTAGATCTACACTCATTGA | 22 | 427.10 | 589.58 | 698.31 | 999.15 |
| bmo-miR-279b* | ATGGGTATAGGTCTAGTAT | 19 | 9.91 | 10.03 | 15.95 | 15.51 |
| bmo-miR-279c | TGACTAGATCCATACTCGTCTG | 22 | 510.64 | 653.50 | 733.45 | 955.67 |
| bmo-miR-279c* | GGGCGAGTTTGCTTCTGGTTCA | 22 | 0.94 | 2.51 | 3.49 | 2.36 |
| bmo-miR-279d | TGACTAGATTTTCACTTATCCT | 22 | 3010.49 | 4156.72 | 4361.85 | 6821.83 |
| bmo-miR-279d* | GATGAGTGACCGTTTAGTTCAA | 22 | 10.62 | 12.39 | 15.20 | 11.12 |
| bmo-miR-2801 | GTGGAATCTTGTGACTTTGTAAAG | 24 | 0.24 | 0.42 | 0.75 | 0.67 |
| bmo-miR-2804 | TTTGCATTGTAATACACTGTTA | 22 | 11.09 | 9.61 | 9.97 | 23.26 |
| bmo-miR-2805 | TTCCTGACGAACCACGGAAA | 20 | 0.00 | 0.56 | 0.25 | 0.00 |
| bmo-miR-2807a | TTCCAGCTGCATACAAATTTCA | 22 | 2.12 | 2.09 | 1.00 | 3.37 |
| bmo-miR-2807b | CAACTGTTTCAACTCCATACAA | 22 | 0.00 | 0.00 | 0.25 | 0.00 |
| bmo-miR-2807c | TACGACGATGCGACAAATATGACA | 24 | 1.42 | 2.09 | 1.50 | 2.70 |
| bmo-miR-2807c* | TCATATTTCGCATTGTTGACG | 21 | 0.47 | 0.97 | 2.24 | 2.02 |
| bmo-miR-2808a | CGTAGAATCTACCATCGGATCG | 22 | 2.83 | 3.76 | 4.24 | 5.39 |
| bmo-miR-2808a* | CGGTGGTAGATTCTGCGAAGTACG | 24 | 3.30 | 5.01 | 3.74 | 6.40 |
| bmo-miR-2808b | CGTAGAATCTACCATCGGATC | 21 | 1.65 | 2.65 | 2.99 | 2.36 |
| bmo-miR-2808c | TGGTGGTAGATTCAGCGAAACA | 22 | 0.00 | 0.28 | 0.00 | 0.00 |
| bmo-miR-2809 | GAAACAGCGAACGGATCACCTGAT | 24 | 1.42 | 2.09 | 1.50 | 2.02 |
| bmo-miR-281 | ACTGTCATGGAGTTGCTCTCTT | 22 | 246.59 | 327.37 | 307.54 | 492.50 |
| bmo-miR-281* | AAGAGAGCTATCCGTCGACAGT | 22 | 6610.67 | 10965.99 | 8360.09 | 13518.26 |
| bmo-miR-2810 | AGATTTTCGAGAACAGCTAATT | 22 | 0.00 | 0.00 | 0.25 | 0.00 |
| bmo-miR-2813 | TTCTAGATTAAGCCGTTGGAA | 21 | 0.24 | 0.00 | 0.00 | 0.34 |
| bmo-miR-2814 | TACAAATTCTGTGGTAGTAGGT | 22 | 0.00 | 0.00 | 0.00 | 0.34 |
| bmo-miR-2817 | AACAAGACTGCGTAGACCGAG | 21 | 1.18 | 1.53 | 1.50 | 1.01 |
| bmo-miR-2819 | TCAATGCCTGCTCTATCGGTTC | 22 | 0.94 | 0.84 | 0.25 | 1.69 |
| bmo-miR-282 | ACCTAGCCTCTCCTTGGCTTTGTCTGT | 27 | 10.85 | 21.17 | 12.21 | 27.64 |
| bmo-miR-282* | ACATAGCCTGATAGAGGTTACG | 22 | 0.00 | 0.00 | 0.25 | 0.00 |
| bmo-miR-2822 | AGGGATATTTTACTCCGCTT | 20 | 0.24 | 0.00 | 0.25 | 0.00 |
| bmo-miR-2826 | AAAAGATCGAGGATCCGATATTG | 23 | 0.47 | 3.62 | 1.25 | 2.36 |
| bmo-miR-2827 | CAGACTATCAGTACGTACGCTG | 22 | 0.24 | 0.14 | 0.00 | 0.00 |
| bmo-miR-2828 | ATATTCGATATGTGAACGGTT | 21 | 0.24 | 0.14 | 0.00 | 0.00 |
| bmo-miR-283 | TAAATATCAGCTGGTAATTCT | 21 | 435.60 | 540.01 | 480.75 | 749.70 |
| bmo-miR-283* | CAGGCTATCAGCTGGTATACAG | 22 | 1.65 | 2.23 | 2.24 | 3.37 |
| bmo-miR-2830 | AGCGACTATCTCAGGGCGCGCT | 22 | 0.47 | 0.84 | 1.25 | 0.67 |
| bmo-miR-2832 | TTGGATAGTGCGTTTTGGATGTC | 23 | 0.00 | 0.14 | 0.00 | 0.00 |
| bmo-miR-2833 | AGAGATTGGAAATGTACTTTTTG | 23 | 0.00 | 0.28 | 0.50 | 0.34 |
| bmo-miR-2833b | AGAGATTGGAAATGTACTTTT | 21 | 0.00 | 0.28 | 0.50 | 0.34 |
| bmo-miR-2834 | TAATATCGGGACTAGACGAC | 20 | 0.00 | 0.14 | 0.00 | 0.34 |
| bmo-miR-2835 | TCACACATTATGAGCTTTAGGA | 22 | 1.18 | 0.56 | 1.25 | 2.02 |
| bmo-miR-2836 | CATCCCGCTGTAGACGACGC | 20 | 4.96 | 6.96 | 6.23 | 5.39 |
| bmo-miR-2838 | AATTCAGCAAACTCACGGGATAA | 23 | 2.83 | 2.92 | 1.74 | 6.40 |
| bmo-miR-2839 | CGCTGGCGCACTGTTTGACGGT | 22 | 1.42 | 0.97 | 0.50 | 1.35 |
| bmo-miR-2839* | TCAAACAGAGCGCCAGCGCTAT | 22 | 0.24 | 0.00 | 0.25 | 0.00 |
| bmo-miR-2840 | TAGGAACTGGAAGAAGAGGAGG | 22 | 0.00 | 0.14 | 0.00 | 0.00 |
| bmo-miR-2841 | ATCATAGTTAAGAGCTCAAAA | 21 | 0.24 | 0.00 | 0.50 | 0.67 |
| bmo-miR-2842 | TGAAGATCCTCGTACTGGTGGCGC | 24 | 0.24 | 0.14 | 0.00 | 0.00 |
| bmo-miR-2843 | TCTAAGGAAATTAGGTCGGATACA | 24 | 5.90 | 12.95 | 10.22 | 17.19 |
| bmo-miR-2843-1* | TTTGCGACGCTGTCTTCGTGATCAA | 25 | 0.47 | 0.28 | 0.25 | 0.00 |
| bmo-miR-2843-2* | TTCGTGATCAAGCCTGACCCCTTAAT | 26 | 2.12 | 0.14 | 0.00 | 1.01 |
| bmo-miR-2845 | CCGTTGCCAGCTGCTGTGCGTA | 22 | 0.00 | 0.28 | 0.00 | 0.34 |
| bmo-miR-2846 | GGGATTACTAATGGCGTAGTGCG | 23 | 1.89 | 2.65 | 1.50 | 2.36 |
| bmo-miR-2847 | TATTGTGTTTGTCAGTGCGGTATA | 24 | 0.47 | 1.67 | 0.75 | 0.67 |
| bmo-miR-2848 | TTCGCGGCAGAAATAGGCAAA | 21 | 0.24 | 0.00 | 0.00 | 0.67 |
| bmo-miR-2849 | AACTTTACGATTATGGACTCAG | 22 | 0.94 | 0.84 | 1.25 | 2.02 |
| bmo-miR-285 | TAGCACCATTCGAATTCAGTGC | 22 | 0.47 | 0.70 | 1.74 | 0.34 |
| bmo-miR-2851 | TGTGATGACTGTATTAGAGAGA | 22 | 0.94 | 0.84 | 0.50 | 1.69 |
| bmo-miR-2854 | TCTTTCAAAACTGCAGGACGG | 21 | 0.00 | 0.14 | 0.00 | 0.67 |
| bmo-miR-2855 | CGGGTAACTTTGCATCGCTGGCA | 23 | 0.00 | 0.14 | 0.00 | 0.34 |
| bmo-miR-2856 | ACATTCGAGAACCGTAAGACAA | 22 | 0.47 | 0.00 | 0.00 | 0.00 |
| bmo-miR-2860 | CGCTGTATGATTGTCCGAGGTA | 22 | 0.94 | 0.28 | 0.25 | 0.00 |
| bmo-miR-2998 | AAGAACAGGATGAGGTAGATAAA | 23 | 8.73 | 12.53 | 14.45 | 17.19 |
| bmo-miR-2999 | CTGCGACGGACTAGACGCGCA | 21 | 5.43 | 6.41 | 1.99 | 1.69 |
| bmo-miR-2a | TATCACAGCCAGCTTTGATGAGC | 23 | 92.50 | 121.84 | 89.47 | 80.90 |
| bmo-miR-2a-1* | GCATCAAAGTCGGTTTGTCATA | 22 | 4.48 | 7.38 | 7.98 | 11.80 |
| bmo-miR-2a-2* | CTCACAAAGTGGTTGTCGTATG | 22 | 0.94 | 0.56 | 0.25 | 1.01 |
| bmo-miR-2b | TATCACAGCCAGCTTTGTTGAGT | 23 | 76.22 | 112.93 | 116.14 | 131.47 |
| bmo-miR-2b* | TCGACAAGGTGGTTGTGACATG | 22 | 1.42 | 1.25 | 1.74 | 1.35 |
| bmo-miR-3000 | CTGCGCTTAGATGAAGACACTA | 22 | 0.24 | 0.14 | 1.00 | 0.34 |
| bmo-miR-3001 | TAAGTTGAAAGAATTGTAGATTTTGA | 26 | 24.54 | 51.94 | 32.40 | 83.26 |
| bmo-miR-305 | ATTGTACTTCATCAGGTGCTCTG | 23 | 33.51 | 37.88 | 77.76 | 46.18 |
| bmo-miR-305* | GGCGCTTGTTGGAGTACACTT | 21 | 0.71 | 1.11 | 0.50 | 0.34 |
| bmo-miR-306a | TCAGGTACTAGGTGACTCTGA | 21 | 4507.72 | 6113.58 | 5554.87 | 7081.06 |
| bmo-miR-306a* | CAGAGCCGCCTCGTGCCTCAG | 21 | 1.18 | 2.23 | 1.50 | 2.02 |
| bmo-miR-306b | TGAGGCACGAGGCGGCTCTGA | 21 | 0.00 | 1.53 | 1.25 | 1.69 |
| bmo-miR-307 | TCACAACCTCCTTGAGTGAG | 20 | 5.43 | 11.98 | 9.47 | 15.51 |
| bmo-miR-308 | AATCACAGGATAATACTGCGAG | 22 | 640.42 | 716.16 | 580.43 | 505.31 |
| bmo-miR-308* | CGTGGTATTATTCTTGTGAATGT | 23 | 10.85 | 15.87 | 12.96 | 13.48 |
| bmo-miR-31 | GGCAAGAAGTCGGCATAGCTG | 21 | 3405.27 | 3974.58 | 2847.34 | 4452.37 |
| bmo-miR-31* | GGCTGTGTCACTTCGAGCCAGC | 22 | 0.00 | 0.42 | 1.00 | 0.67 |
| bmo-miR-316 | ACGGCAAAGTGAAAAGGTCTCC | 22 | 13.45 | 26.04 | 19.94 | 24.95 |
| bmo-miR-316* | TGTCTTTTTCCGCTTTGCTGCTG | 23 | 102.88 | 152.48 | 176.20 | 225.52 |
| bmo-miR-317 | AGTGAACACAGCTGGTGGTATC | 22 | 48.37 | 65.73 | 70.78 | 82.25 |
| bmo-miR-317* | CGGGTGCCACGCTGTGCTCTCT | 22 | 1.89 | 2.23 | 2.24 | 3.03 |
| bmo-miR-3203 | ATGTCAGCTCAGTCAGTACACG | 22 | 1.65 | 0.14 | 0.50 | 0.67 |
| bmo-miR-3203* | TGTATTGAAATAGCTTGACGTG | 22 | 0.00 | 0.00 | 0.25 | 0.00 |
| bmo-miR-3204a | CGCGTCATAAACTTCCCATAT | 21 | 0.47 | 0.70 | 0.75 | 0.34 |
| bmo-miR-3204a* | TATGGGAGTTTATGACTTAGG | 21 | 0.24 | 0.97 | 0.50 | 0.34 |
| bmo-miR-3219 | GTGAGACTAATATATCCATGGTGT | 24 | 5.19 | 9.05 | 9.22 | 9.10 |
| bmo-miR-3220 | TTCTTTCCGCGCGTGACGGTTTGGA | 25 | 0.00 | 0.14 | 0.00 | 0.00 |
| bmo-miR-3228 | GAGATCAAATGTTGCTGTCCTTGGA | 25 | 0.71 | 0.84 | 0.25 | 0.67 |
| bmo-miR-3238 | AAATGAATCATGCATTGACTACATG | 25 | 1.42 | 2.51 | 4.24 | 4.38 |
| bmo-miR-3242 | CCTTTGCCTTCATCTTCTGCAACTT | 25 | 0.00 | 0.14 | 0.00 | 0.00 |
| bmo-miR-3257* | TGGCGATTACGCGATTTGCCGTGACCAGA | 29 | 0.24 | 0.00 | 0.00 | 0.00 |
| bmo-miR-3263 | TAGCTGTTGAGTCCCGTTGAGAA | 23 | 0.00 | 0.14 | 0.00 | 0.00 |
| bmo-miR-3268 | TGAAGTATGATTCATTAGTAGACTA | 25 | 0.00 | 0.14 | 0.00 | 0.00 |
| bmo-miR-3268* | GTGTTTGTCGATGAATTATTGGTACTCA | 28 | 0.47 | 0.00 | 0.00 | 0.00 |
| bmo-miR-3271 | TGACATCATGTGCGCGTGCTGCT | 23 | 1.18 | 0.70 | 0.25 | 1.35 |
| bmo-miR-3274 | TCGATATACTTGCGTCTCCATGCA | 24 | 0.00 | 0.14 | 0.00 | 0.00 |
| bmo-miR-3275 | TGATTCTTGAGTAGGACTTCCTTC | 24 | 0.47 | 1.25 | 0.00 | 0.34 |
| bmo-miR-3281 | ATCTTATGTCGGTGTGGCGTGT | 22 | 0.00 | 0.00 | 0.25 | 0.00 |
| bmo-miR-3286 | TTGTGCGTGTTCCAATAGTTAT | 22 | 0.00 | 0.14 | 0.00 | 0.00 |
| bmo-miR-33 | GTGCATTGTAGTTGCATTGCA | 21 | 25.25 | 27.99 | 31.65 | 43.49 |
| bmo-miR-33* | CAATATGACTACAAGGCAAATC | 22 | 21.00 | 24.09 | 28.16 | 47.87 |
| bmo-miR-3304a | TAAACAGCTTGGAATATTTACAG | 23 | 0.71 | 0.14 | 0.50 | 0.34 |
| bmo-miR-3305 | TTACATATGGTTATTACGTCACTAG | 25 | 0.00 | 0.00 | 0.25 | 0.34 |
| bmo-miR-3306 | TAATCGATAGATTTTGACGAGAGC | 24 | 0.24 | 0.56 | 1.25 | 0.67 |
| bmo-miR-3308 | TACTTTATCTTGGTAATTGGTTGTC | 25 | 0.24 | 0.28 | 0.00 | 0.00 |
| bmo-miR-3309 | TGTAATAGTATAATTGTGCTCTTG | 24 | 0.24 | 0.00 | 0.25 | 0.00 |
| bmo-miR-3312 | TCCTTTCTTGCTTCGCTTGCGTG | 23 | 0.00 | 0.14 | 0.00 | 0.34 |
| bmo-miR-3316 | TGCATTACTGTTTCTTCGACTCCG | 24 | 0.00 | 0.28 | 0.00 | 0.00 |
| bmo-miR-3321 | CAGAAGTCAAGCATCGCCTTCCA | 23 | 0.24 | 0.00 | 0.25 | 0.00 |
| bmo-miR-3323 | CAATACGAATAATGTTAATGCCT | 23 | 0.94 | 1.11 | 0.75 | 0.67 |
| bmo-miR-3324 | TCCATGATCCCTGAGTGCCTAGGT | 24 | 0.47 | 0.00 | 0.00 | 0.34 |
| bmo-miR-3326 | TAGGTTTTGTGGTTCTTGTGAAATA | 25 | 0.47 | 0.42 | 0.25 | 0.34 |
| bmo-miR-3327* | ATATGTAACGTTTTTGTTGTCCT | 23 | 0.00 | 0.14 | 0.25 | 0.00 |
| bmo-miR-3328 | TGTTTGTGCACTTCAGGGAATCTAT | 25 | 0.00 | 0.00 | 0.00 | 0.34 |
| bmo-miR-3329 | GCATACAATAATTTATGACAGAT | 23 | 0.00 | 0.00 | 0.25 | 0.00 |
| bmo-miR-3330 | TACCGTAGTGAAAGCGCTCCACGA | 24 | 0.00 | 0.00 | 0.00 | 0.34 |
| bmo-miR-3332 | TCCTCGCCATGCCACCGCCGCTTCA | 25 | 0.24 | 0.00 | 0.00 | 0.00 |
| bmo-miR-3333 | CCGTTCGAGAAGCAAGACAAAGTG | 24 | 1.18 | 0.84 | 0.00 | 1.01 |
| bmo-miR-3334 | TGAACCAGAATGATGGAAGGACAG | 24 | 8.73 | 10.03 | 12.46 | 15.17 |
| bmo-miR-3337 | TCCATATCCGTGCTCGGACGCTT | 23 | 0.24 | 0.56 | 0.25 | 0.34 |
| bmo-miR-3338 | ATGTACTTACTTTGTTTGTTCT | 22 | 0.24 | 0.56 | 0.00 | 0.34 |
| bmo-miR-3339 | TAGACTTTTTAGATGTTGGTCAA | 23 | 0.24 | 0.00 | 0.00 | 0.34 |
| bmo-miR-3340 | TATACGGATGCGATGACGATCGGCT | 25 | 21.47 | 28.55 | 20.69 | 33.04 |
| bmo-miR-3343 | TCTCCATCGTTCCTGCACCGTAGC | 24 | 0.00 | 0.28 | 0.00 | 0.34 |
| bmo-miR-3344 | TTGCAAGAAGGACTCAGCCAGCGAG | 25 | 0.00 | 0.00 | 0.00 | 0.34 |
| bmo-miR-3345 | TATCGTTACGCGCGTCGCTTAGG | 23 | 0.47 | 0.70 | 0.25 | 0.67 |
| bmo-miR-3350 | TCATGTATTCTAATTTTTCTTTATA | 25 | 0.00 | 0.00 | 0.25 | 1.01 |
| bmo-miR-3351 | TTACGTTGTGGATGTCTATGGGC | 23 | 2.12 | 2.37 | 1.74 | 2.36 |
| bmo-miR-3354 | TCACATCAGGCACAGTTACAGGGG | 24 | 1.89 | 2.78 | 1.25 | 1.69 |
| bmo-miR-3355 | TAATACTATAAAGGTTCAAGTCA | 23 | 0.00 | 0.14 | 0.25 | 0.00 |
| bmo-miR-3362 | TGATTTGAAGTTTATGAATGTTGTA | 25 | 0.24 | 0.70 | 1.00 | 1.01 |
| bmo-miR-3363 | TCATACGTATTTGTGTACTGCGTTT | 25 | 0.00 | 0.14 | 0.25 | 0.67 |
| bmo-miR-3372* | TTTATTATGTTCTTGGCGTCAGAGCCAAA | 29 | 0.00 | 0.14 | 0.00 | 0.34 |
| bmo-miR-3373* | GCCACGTGACATTCCCGCAGCTTC | 24 | 0.00 | 0.14 | 0.00 | 0.00 |
| bmo-miR-3374 | TGCTTTATTTGCTCGCACTCAAC | 23 | 0.24 | 0.28 | 0.25 | 0.00 |
| bmo-miR-3374* | TGAAGATGCACCAGATGTTGAGAGGCC | 27 | 0.00 | 0.14 | 0.00 | 0.00 |
| bmo-miR-3375 | TGCTATCTTTGAAAAGTTTGGAATA | 25 | 0.24 | 0.28 | 0.25 | 0.00 |
| bmo-miR-3377 | TGAAGTTATGAAGTCAGGATA | 21 | 0.24 | 0.14 | 0.25 | 0.34 |
| bmo-miR-3378 | TTCCATGTTGATCGGGGCTGCTTC | 24 | 0.47 | 0.14 | 0.25 | 0.00 |
| bmo-miR-3378* | TTGCTGCTCCAATTGTCTGGACACG | 25 | 0.00 | 0.42 | 0.25 | 0.00 |
| bmo-miR-3379 | AAATCTGACCGAAGACTACGA | 21 | 8.97 | 8.08 | 3.49 | 2.70 |
| bmo-miR-3379* | CGTACTCGTCGTTACGTGTTG | 21 | 2.60 | 2.78 | 1.25 | 0.67 |
| bmo-miR-3381 | TCAAGATTCCTCGCAAAGGTTCAC | 24 | 0.00 | 0.14 | 0.25 | 0.34 |
| bmo-miR-3382 | TCAAGTTTTGTGTTCTGGGTCGT | 23 | 0.47 | 0.28 | 0.00 | 0.00 |
| bmo-miR-3383 | CCACGAAGTTGACGCGCTGTCTCT | 24 | 0.00 | 0.00 | 0.00 | 0.34 |
| bmo-miR-3387 | TTCAGGTTCGTGCATAATGTGCT | 23 | 0.24 | 0.28 | 0.50 | 0.34 |
| bmo-miR-3387* | ACATTAATGAAAACGATCGTG | 21 | 0.47 | 1.53 | 1.74 | 2.02 |
| bmo-miR-3391 | TGACAAAAAGTGATGTGAGTCCGTC | 25 | 0.24 | 0.00 | 0.00 | 1.01 |
| bmo-miR-3395 | AGCTAGTTCGGCGATGTCGCTCT | 23 | 0.00 | 0.14 | 0.00 | 0.00 |
| bmo-miR-3399 | TATCTACATTGAATACCTGGCTATG | 25 | 0.24 | 0.00 | 0.25 | 0.00 |
| bmo-miR-34 | TGGCAGTGTGGTTAGCTGGTTG | 22 | 40.82 | 68.65 | 84.98 | 110.23 |
| bmo-miR-34* | AGCCACTAACGACACTGCTCCT | 22 | 4.96 | 3.90 | 7.48 | 7.08 |
| bmo-miR-3403 | TGAATCTAGTCTGAAGTCTGATTTG | 25 | 0.24 | 0.14 | 0.00 | 0.00 |
| bmo-miR-3404 | TGAATCTTTTCTGCAATAGTGTGAC | 25 | 0.00 | 0.28 | 0.25 | 1.01 |
| bmo-miR-3405 | TCCATTGTGTTCTTGAAACTCTCTA | 25 | 2.12 | 3.90 | 2.24 | 4.38 |
| bmo-miR-3406 | TAGTAAGTAGCACGTTTGATGAGCA | 25 | 0.00 | 0.00 | 0.50 | 0.00 |
| bmo-miR-3414 | TCCTTAAGTTTTTTGAACGCAGTG | 24 | 0.00 | 0.14 | 0.00 | 0.00 |
| bmo-miR-375 | ACCCGAGCGGTCTGAGCAAACT | 22 | 1.42 | 2.23 | 0.50 | 0.34 |
| bmo-miR-375* | TTTGTTCGCCCCGGCTCGTGTCG | 23 | 0.24 | 1.11 | 0.25 | 0.34 |
| bmo-miR-7 | TGGAAGACTAGTGATTTTGTTGT | 23 | 15.10 | 19.77 | 20.69 | 19.89 |
| bmo-miR-7* | AAGAAATCACTAATCTGCCTA | 21 | 6.14 | 7.38 | 6.98 | 5.06 |
| bmo-miR-71 | TGAAAGACATGGGTAGTGA | 19 | 0.24 | 0.28 | 0.25 | 0.67 |
| bmo-miR-71* | TTCTCACTACCTTGTCTTTCAT | 22 | 49.32 | 80.21 | 71.03 | 85.96 |
| bmo-miR-745 | CAGCTGCCTAGCGAAGGGCAACG | 23 | 25.48 | 24.23 | 16.70 | 46.52 |
| bmo-miR-745* | CGGCTCATCGTGTGGCAGTTTGCT | 24 | 4.01 | 6.41 | 3.99 | 11.12 |
| bmo-miR-750 | CCAGATCTATCTTTCCAGCT | 20 | 13858.22 | 15899.02 | 16223.23 | 21895.11 |
| bmo-miR-750* | AGTTGGACAGGGGATCTTGACA | 22 | 95.57 | 133.68 | 139.06 | 156.08 |
| bmo-miR-79 | TTCATAAAGCTAGATTACCAAAGCAT | 26 | 607.38 | 749.86 | 700.56 | 906.12 |
| bmo-miR-79* | CTTTGGCGATTTAGCTCCGTGA | 22 | 1.89 | 4.18 | 2.49 | 3.37 |
| bmo-miR-8 | TAATACTGTCAGGTAAAGATGTC | 23 | 2747.39 | 3487.63 | 2800.49 | 3853.69 |
| bmo-miR-8* | CATCTTACCGGGCAGCATTAGA | 22 | 37.05 | 38.15 | 28.66 | 24.27 |
| bmo-miR-87 | TGAGCAAACTTTCAGGTGTGT | 21 | 22.42 | 41.22 | 38.88 | 28.99 |
| bmo-miR-927 | TTTAGAATTCCTACGCTTTACC | 22 | 0.24 | 1.95 | 1.74 | 3.71 |
| bmo-miR-927* | CAAAGCGTTTGGATTCTAA | 19 | 0.00 | 0.28 | 0.50 | 0.67 |
| bmo-miR-929 | ATTGACTCTAGTAGGGAGTCC | 21 | 0.00 | 0.70 | 1.50 | 0.67 |
| bmo-miR-929* | CTCCCTAATCGAGTCAGGTTGA | 22 | 0.47 | 0.56 | 1.25 | 0.34 |
| bmo-miR-92a | TATTGCACCAGTCCCGGCCTAT | 22 | 4.48 | 8.63 | 13.46 | 11.46 |
| bmo-miR-92b | AATTGCACCAATCCCGGCCTGC | 22 | 27.84 | 37.74 | 44.86 | 46.18 |
| bmo-miR-932 | TCAATTCCGTAGTGCATTGCAG | 22 | 0.00 | 0.56 | 0.50 | 0.00 |
| bmo-miR-965 | CGGGAGAAGCTATAGCGCTATATG | 24 | 25.48 | 34.67 | 35.39 | 53.26 |
| bmo-miR-965* | TAAGCGTATAGCTTTTCCCCTT | 22 | 50.50 | 63.08 | 73.02 | 69.44 |
| bmo-miR-970 | TCATAAGACACACGCGGCTCT | 21 | 42.95 | 64.33 | 51.84 | 80.57 |
| bmo-miR-970* | AGCCTTGCGTGTGCTCTTATTGGTA | 25 | 0.71 | 1.11 | 0.75 | 1.35 |
| bmo-miR-989 | GTGTGATGTGACGTAGTGGAA | 21 | 0.00 | 0.70 | 1.00 | 0.34 |
| bmo-miR-993a | GAAGCTCGTCTCTACAGGTATCT | 23 | 0.24 | 0.42 | 0.00 | 0.34 |
| bmo-miR-993a* | TCTACCCTGTAGATCCGGGCTTTT | 24 | 0.00 | 0.42 | 1.25 | 1.01 |
| bmo-miR-993b | AAAGCTCGTCTCTACAGGTATAT | 23 | 1.18 | 2.51 | 1.74 | 1.69 |
| bmo-miR-993b* | TACCCTGTAGATCCGGGCTTTCG | 23 | 11.56 | 20.89 | 19.94 | 30.34 |
| bmo-miR-998 | TAGCACCATGGGATTCAGCT | 20 | 56.40 | 73.66 | 40.12 | 35.40 |
| bmo-miR-9a | TCTTTGGTTATCTAGCTGTATGA | 23 | 26.19 | 107.50 | 33.15 | 71.80 |
| bmo-miR-9a* | ATAAAGCTAGGTTACCGGAGTTA | 23 | 0.94 | 3.90 | 0.50 | 2.36 |
| bmo-miR-9b | GCTTTGGTAATCTAGCTTTATGA | 23 | 606.68 | 749.44 | 700.06 | 905.78 |
| bmo-miR-9b* | ACGGAGCTAAATCGCCAAAGCG | 22 | 1.89 | 4.18 | 2.49 | 3.37 |
| bmo-miR-9c | TAAAGTTATGGTACCGAAGTTA | 22 | 176.03 | 222.52 | 230.78 | 276.42 |
| bmo-miR-9c* | TCTTTGGTATCCTAGCTG | 18 | 232.67 | 371.66 | 338.69 | 372.83 |
| bmo-miR-9d | ATAAAGCTAATTCACTGAGTGT | 22 | 0.24 | 0.56 | 0.50 | 1.35 |
| bmo-miR-iab-4-5p | ACGTATACTGAATGTATCCTGA | 22 | 11.80 | 15.04 | 4.74 | 14.50 |
