# Supplementary material for: Involvement of MicroRNAs in Infection of Silkworm with Bombyx mori Cytoplasmic Polyhedrosis Virus (BmCPV)
Source: PLoS One. 2013 Jul 2;8(7):e68209. doi: 10.1371/journal.pone.0068209 (PMC3699532; doi:10.1371/journal.pone.0068209)
Supplement: Table S2 — Known miRNAs with abundant expression in midgut in 4 small RNA libraries. Known silkworm miRNAs or MiR family with total sequence counts >104 in each sample. (DOC) [file pone.0068209.s003.doc]

Table S2 Known miRNAs with abundant expression in midgut in 4 small RNA libraries

| MicroRNA | Normalization Counts（Transcripts per million,TPM) | | | |
| --- | --- | --- | --- | --- |
|  | 72t | 72c | 96t | 96c |
| bmo-bantam | 3333.77 | 5343.54 | 4459.29 | 5199.72 |
| bmo-let-7 | 459.67 | 620.21 | 573.46 | 695.77 |
| bmo-miR-10 | 9889.22 | 14757.04 | 7269.75 | 11383.76 |
| bmo-miR-100 | 1954.30 | 2293.99 | 1606.97 | 1847.63 |
| bmo-miR-11 | 811.73 | 982.40 | 827.16 | 757.12 |
| bmo-miR-1175-3p | 2212.21 | 2760.47 | 2950.02 | 3906.28 |
| bmo-miR-1175-5p | 965.35 | 987.14 | 1257.32 | 1222.65 |
| bmo-miR-12 | 1751.13 | 1798.26 | 1894.32 | 2268.66 |
| bmo-miR-14 | 649.86 | 1042.14 | 1205.48 | 1056.12 |
| bmo-miR-184 | 2531.00 | 3165.82 | 2173.45 | 3210.17 |
| bmo-miR-1a | 420.73 | 693.74 | 324.48 | 581.15 |
| bmo-miR-263a | 8841.05 | 9211.73 | 4804.46 | 5140.05 |
| bmo-miR-263a* | 8.73 | 9.19 | 9.47 | 10.11 |
| bmo-miR-263b | 4.01 | 5.01 | 2.74 | 2.36 |
| bmo-miR-263b* | 0.24 | 0.97 | 0.75 | 0.67 |
| bmo-miR-2758 | 1325.20 | 1811.35 | 1617.94 | 1905.61 |
| bmo-miR-2766 | 986.35 | 1353.78 | 1091.58 | 1405.69 |
| bmo-miR-2766* | 968.89 | 1432.87 | 1596.01 | 1793.69 |
| bmo-miR-2778a | 833.91 | 1228.04 | 1141.93 | 1308.95 |
| bmo-miR-2778a* | 17.70 | 29.52 | 47.85 | 52.59 |
| bmo-miR-2778a-2* | 0.00 | 0.14 | 0.50 | 0.00 |
| bmo-miR-2778a-4* | 0.47 | 0.70 | 0.25 | 1.01 |
| bmo-miR-2778b | 20.53 | 33.00 | 17.45 | 43.15 |
| bmo-miR-2778b* | 17.70 | 29.52 | 47.85 | 52.59 |
| bmo-miR-2778c | 17.70 | 29.52 | 47.85 | 52.59 |
| bmo-miR-2778c* | 0.47 | 2.65 | 3.74 | 4.38 |
| bmo-miR-2778d | 17.70 | 29.52 | 47.85 | 52.59 |
| bmo-miR-2778d* | 1.65 | 5.01 | 3.99 | 2.36 |
| bmo-miR-278* | 1053.13 | 1186.40 | 1950.15 | 1350.07 |
| bmo-miR-279a | 82.02 | 66.54 | 119.33 | 82.02 |
| bmo-miR-279b | 589.58 | 698.31 | 999.15 | 589.58 |
| bmo-miR-279b* | 10.03 | 15.95 | 15.51 | 10.03 |
| bmo-miR-279c | 653.50 | 733.45 | 955.67 | 653.50 |
| bmo-miR-279c* | 2.51 | 3.49 | 2.36 | 2.51 |
| bmo-miR-279d | 4156.72 | 4361.85 | 6821.83 | 4156.72 |
| bmo-miR-279d* | 12.39 | 15.20 | 11.12 | 12.39 |
| bmo-miR-281 | 246.59 | 327.37 | 307.54 | 492.50 |
| bmo-miR-281* | 6610.67 | 10965.99 | 8360.09 | 13518.26 |
| bmo-miR-283 | 435.60 | 540.01 | 480.75 | 749.70 |
| bmo-miR-306a | 4507.72 | 6113.58 | 5554.87 | 7081.06 |
| bmo-miR-308 | 640.42 | 716.16 | 580.43 | 505.31 |
| bmo-miR-31 | 3405.27 | 3974.58 | 2847.34 | 4452.37 |
| bmo-miR-750 | 13858.22 | 15899.02 | 16223.23 | 21895.11 |
| bmo-miR-79 | 607.38 | 749.86 | 700.56 | 906.12 |
| bmo-miR-8 | 2747.39 | 3487.63 | 2800.49 | 3853.69 |
| bmo-miR-9a | 26.19 | 107.50 | 33.15 | 71.80 |
| bmo-miR-9a* | 0.94 | 3.90 | 0.50 | 2.36 |
| bmo-miR-9b | 606.68 | 749.44 | 700.06 | 905.78 |
| bmo-miR-9b* | 1.89 | 4.18 | 2.49 | 3.37 |
| bmo-miR-9c | 176.03 | 222.52 | 230.78 | 276.42 |
| bmo-miR-9c* | 232.67 | 371.66 | 338.69 | 372.83 |
| bmo-miR-9d | 0.24 | 0.56 | 0.50 | 1.35 |
