# Supplementary material for: Involvement of MicroRNAs in Infection of Silkworm with Bombyx mori Cytoplasmic Polyhedrosis Virus (BmCPV)
Source: PLoS One. 2013 Jul 2;8(7):e68209. doi: 10.1371/journal.pone.0068209 (PMC3699532; doi:10.1371/journal.pone.0068209)
Supplement: Table S3 — Conserved miRNAs in 4 small RNA libraries. Frequency of conserved miRNAs in each sample. (DOC) [file pone.0068209.s004.doc]

Table S3 Conserved microRNAs in 4 small RNA libraries

| MicroRNA | Sequence | Length | Normalization Counts  （Transcripts per million,TPM) | | | |
| --- | --- | --- | --- | --- | --- | --- |
|  |  |  | 72t | 72c | 96t | 96c |
| miR-2478 | ATCCCACTTCTGACACCA | 18 | 126.95 | 99.84 | 161.00 | 86.97 |
| miR-981 | TTCGTTGTCGTCGAAACCT | 19 | 6.37 | 8.22 | 9.72 | 10.79 |
| miR-3351 | TTACGTTGTAGATGCCTATG | 20 | 0.94 | 3.76 | 0.75 | 2.70 |
| miR-1692 | GATAGCTCAGTTGGTAGAG | 19 | 1.65 | 1.95 | 1.25 | 0.67 |
| miR-2774 | CTCGATGGAAGATCTTCCCT | 20 | 0.94 | 2.37 | 1.00 | 1.01 |
