# Supplementary material for: Involvement of MicroRNAs in Infection of Silkworm with Bombyx mori Cytoplasmic Polyhedrosis Virus (BmCPV)
Source: PLoS One. 2013 Jul 2;8(7):e68209. doi: 10.1371/journal.pone.0068209 (PMC3699532; doi:10.1371/journal.pone.0068209)
Supplement: Table S4 — Novel miRNAs in 4 small RNA libraries. Frequency of novel miRNAs in each sample. (DOC) [file pone.0068209.s005.doc]

Table S4 Novel microRNAs in 4 small RNA libraries

| MicroRNA | Sequence | Normalization Counts  （Transcripts per million,TPM) | | | |
| --- | --- | --- | --- | --- | --- |
|  |  | 72t | 72c | 96t | 96c |
| Novel-1 | GTGATCCGTCGCGTTGACGCG | 8.26 | 12.39 | 10.72 | 11.12 |
| Novel-2 | ACCCGTTCGTCGTGGATTTAAGACG | 118.69 | 117.39 | 165.48 | 67.42 |
| Novel-3 | TACTTTGATCAATATGTTCAGT | 5.90 | 11.00 | 10.22 | 9.10 |
| Novel-60*-2 | AGTTCTCGTGTATATGAATT | 0.47 | 0.84 | 0.25 | 0.34 |
| Novel-4 | TGAAACAATCAAACGGACGAAT | 81.65 | 116.41 | 87.97 | 128.10 |
| Novel-5 | ATTAGGATTTTTGTTAAACC | 125.06 | 106.80 | 39.63 | 148.32 |
| Novel-6 | ATTTGGATCGCGGAGATC | 118.69 | 84.11 | 139.31 | 66.75 |
| Novel-7 | CGTACGAGAGGAGGCATAGT | 5.19 | 10.44 | 8.22 | 9.44 |
| Novel-58* | AACTTGATCATTTAGAGGAAGT | 107.84 | 73.38 | 131.09 | 60.00 |
| Novel-60*-1 | CGAGTTCTCGTGTATATG | 0.47 | 0.42 | 0.25 | 0.67 |
| Novel-45* | CTTTACTAGTCGCGTTTCGC | 11.80 | 6.68 | 7.73 | 3.71 |
| Novel-8 | ATTTTACATCCCGCTGTAGA | 4.48 | 6.96 | 8.97 | 9.44 |
| Novel-9 | CGCGGATGCGGGCTTGGGAC | 5.43 | 8.49 | 6.23 | 7.42 |
| Novel-50* | TTACTTACTCGGTTGGGCGGAA | 109.96 | 93.58 | 26.67 | 72.81 |
| Novel-24* | CGTGAACGTTTAACGTCCCGAGAA | 116.10 | 71.85 | 73.52 | 45.85 |
| Novel-10 | TTCGGAACGCGAAGAGCACC | 117.51 | 58.07 | 90.47 | 48.54 |
| Novel-11 | GTTGTTGGGAAGTTGACC | 179.10 | 29.38 | 72.52 | 48.54 |
| Novel-12 | ATTCACTGTAGGTATAGATA | 0.71 | 2.37 | 6.73 | 23.60 |
| Novel-30* | AACGCCCTTTGAGCGAAAGGG | 8.26 | 4.18 | 10.22 | 3.37 |
| Novel-13 | CGTTATGTTCGTTTTAGC | 556.65 | 616.32 | 851.58 | 1123.21 |
| Novel-14 | ACGACATGGTGTTGCTGAGCCT | 4.72 | 6.68 | 3.99 | 8.09 |
| Novel-15 | TATCGAGAGCGTTAAGAAAC | 3.78 | 5.43 | 4.98 | 6.07 |
| Novel-16 | TCACGAATTGCTCTGCAGAACCT | 3.54 | 5.85 | 4.98 | 4.38 |
| Novel-32* | CGGGAGGTCATCTTGGAC | 0.47 | 0.28 | 0.25 | 0.00 |
| Novel-17 | ATTCGAGAACGTCGTCTGGCG | 6.14 | 3.76 | 7.23 | 2.36 |
| Novel-17* | CGCGAGCCGTCGCTTGTA | 0.71 | 0.00 | 0.25 | 0.34 |
| Novel-18 | TATTATAAAAGTTGTAGGCT | 5.43 | 5.15 | 2.74 | 4.05 |
| Novel-2* | GATCCACGCATTCGGACG | 59.70 | 42.05 | 77.01 | 46.18 |
| Novel-19 | TTCCTGTGCTCGAGCTACTTGGATA | 2.12 | 4.46 | 5.98 | 5.06 |
| Novel-20 | AACGTGAAGAACCTGAAAGCT | 2.12 | 4.32 | 4.49 | 6.40 |
| Novel-21 | ATGGCTTGTCGTTGCGAT | 4.25 | 2.92 | 7.23 | 2.36 |
| Novel-22 | TAATGTCAATAGTATAGA | 2.60 | 3.90 | 3.24 | 6.07 |
| Novel-23 | CGAGGACCATGGTGGACTG | 3.07 | 3.34 | 3.24 | 6.40 |
| Novel-24 | TTTCTCTCGGGCGTACGTTTAC | 2792.93 | 7260.44 | 844.36 | 2811.72 |
| Novel-4* | GTTCGTTCGGTTAGTTCATT | 3.78 | 4.18 | 3.24 | 2.70 |
| Novel-25 | AAACACGATCACGCCGTACG | 3.78 | 4.04 | 3.99 | 1.69 |
| Novel-26 | CGGATAGGCCACATACTGTC | 1.89 | 3.20 | 5.48 | 4.38 |
| Novel-27 | GAATCCCAGGCATGTACCAA | 3.54 | 4.18 | 2.99 | 3.03 |
| Novel-55* | CTCAAAATTATCTTCTTTCAG | 0.00 | 0.56 | 0.00 | 0.00 |
| Novel-28 | CAATTCTCATTTCGGGCGTC | 0.94 | 5.15 | 1.99 | 5.06 |
| Novel-29 | AAAATCGGAAACAGAATTGCTC | 3.07 | 3.20 | 2.99 | 4.38 |
| Novel-30 | AGAGATCTTATGTCGATGTGGCG | 39.88 | 36.48 | 64.05 | 28.65 |
| Novel-31 | AGCCGTAACGAGTAGGACG | 44.83 | 35.09 | 55.58 | 34.05 |
| Novel-32 | ATGTTAACAAGCTGAACCT | 3.30 | 2.09 | 4.98 | 3.71 |
| Novel-33 | ATTTTCAGGAAGTTCACT | 8.02 | 1.95 | 1.74 | 1.69 |
| Novel-47* | ATCAGCGGTGGTCTGGGGTACC | 29.50 | 62.38 | 22.93 | 31.35 |
| Novel-34 | TCGAGAACGTTACATAACTC | 3.07 | 2.92 | 2.49 | 4.72 |
| Novel-35 | AGGCAGTCGCTGACTTGGTT | 1.42 | 2.78 | 5.73 | 2.70 |
| Novel-31* | CTATACACTACCGTTACCGGC | 3.30 | 2.09 | 6.23 | 1.01 |
| Novel-36 | CTTGGGTCGTAAAAGCAACT | 2.83 | 2.65 | 2.99 | 4.38 |
| Novel-37 | CTGTCTACCTGACGGACT | 2.12 | 4.04 | 3.24 | 1.69 |
| Novel-54* | CACTACCGTTACCGGCACG | 4.01 | 1.67 | 5.48 | 1.69 |
| Novel-38 | TAGACCTGGCACTGCTGAGG | 2.60 | 3.76 | 1.50 | 3.71 |
| Novel-39 | TATTCCAAAGCCACTAGTTGGGCG | 1.18 | 3.20 | 5.23 | 2.02 |
| Novel-40 | AGTAGATACTAGTAGATACT | 3.07 | 3.48 | 1.50 | 3.71 |
| Novel-41 | TGATCAACGCAAAGTCGCCA | 5.19 | 2.09 | 1.74 | 3.03 |
| Novel-42 | CGATTCTGACGTGCAAATC | 49.79 | 30.77 | 43.86 | 27.98 |
| Novel-43 | CTGGTGTAGTGGTAAGTGACT | 2.36 | 3.62 | 3.49 | 1.01 |
| Novel-44 | TGGCGTCGCGACCTTGGGTC | 3.07 | 3.62 | 1.00 | 2.70 |
| Novel-52* | CGAGTGCGACGCGTCTGTTGT | 52.15 | 28.55 | 36.64 | 24.27 |
| Novel-45 | ACGCGAGACGCGACGTCGAAGC | 42.24 | 22.56 | 53.33 | 18.88 |
| Novel-46 | GAATCAGCATGTTCTCCCT | 72.21 | 10.86 | 23.68 | 30.00 |
| Novel-8* | TTTGAGTGCGTGGGAAGTTG | 0.00 | 0.14 | 0.25 | 0.00 |
| Novel-47 | TATTCGAGACCTCTGCTGATCCT | 11650.97 | 12891.25 | 15102.49 | 16854.50 |
| Novel-48 | CGCAACTCACTGACGACGTATT | 21.47 | 33.28 | 23.68 | 40.45 |
| Novel-49 | CGCGTGAACAGTAGTTGCTCGC | 30.20 | 22.28 | 45.11 | 21.24 |
| Novel-50 | CGGTGTTTCGTTCCAAGCGTGCAGA | 656.23 | 207.48 | 147.04 | 388.34 |
| Novel-51 | TGGGTGAGAACTCCGGCT | 33.04 | 20.47 | 40.62 | 22.59 |
| Novel-52 | ATACAGTTTCGGGCACTC | 312.89 | 295.07 | 431.65 | 166.53 |
| Novel-48* | AGGCGTCGTTATTTAGGTG | 2.60 | 1.95 | 2.24 | 0.67 |
| Novel-51* | AGCCGGAGATCTGATGAC | 36.58 | 19.63 | 25.17 | 17.19 |
| Novel-53 | AGGATTGTGGGTGGTTCTGCC | 11.80 | 26.46 | 10.97 | 48.88 |
| Novel-38* | ATCGAAGCAATGTCAGAGCTT | 0.00 | 0.28 | 0.00 | 0.00 |
| Novel-11* | GTGAGGTCTTCGGACCGACA | 39.41 | 16.57 | 14.45 | 13.48 |
| Novel-46* | CCCGGGCAACCCGCTGAAACT | 2.83 | 0.42 | 1.74 | 1.01 |
| Novel-54 | AAGCCGTAACGAGTAGGAC | 16.28 | 14.48 | 26.17 | 16.85 |
| Novel-53* | TGGCAGAAGAGCCCATCGAA | 0.71 | 0.42 | 1.00 | 4.05 |
| Novel-6*-1 | CATCACGGCTCCGAAGGTCCG | 21.95 | 14.90 | 24.67 | 9.44 |
| Novel-55 | GTAAGTAGAAAATTCTGTGTCT | 156.21 | 212.91 | 167.48 | 285.86 |
| Novel-13* | CACTCGCGAAAATCGTCTTAC | 158.81 | 165.01 | 201.87 | 289.23 |
| Novel-14* | ACTCAGCAATGCCAGTGTCGGCT | 0.47 | 1.67 | 0.75 | 0.34 |
| Novel-56 | TTTCATTGTTTCATTACTT | 16.05 | 11.56 | 25.67 | 6.07 |
| Novel-59* | TCAGTTAGCGTCCGTCCGTC | 0.71 | 0.84 | 1.25 | 0.67 |
| Novel-57 | CGCGAAAATCGTCTTACGTTTCG | 6065.11 | 7449.96 | 8728.68 | 9858.06 |
| Novel-58 | TACCGATTGAATGATTTAG | 138.51 | 133.54 | 194.89 | 90.68 |
| Novel-59 | TTCAGTCTGACTGACTGACT | 12.51 | 8.77 | 15.95 | 14.50 |
| Novel-60 | TTCATAATCACGTCGAACCT | 12.03 | 14.48 | 7.98 | 10.45 |
| Novel-42* | CCGAAGTTTCCCTCAGGATAGCTG | 13.21 | 11.28 | 14.70 | 7.42 |
| Novel-61 | TGTTCTACTTTTCTCCCGCGGT | 11.56 | 11.84 | 7.23 | 17.87 |
| Novel-6*-2 | ATCACGGCTCCGAAGGTCCG | 14.16 | 9.19 | 13.71 | 10.11 |
