# Supplementary material for: Involvement of MicroRNAs in Infection of Silkworm with Bombyx mori Cytoplasmic Polyhedrosis Virus (BmCPV)
Source: PLoS One. 2013 Jul 2;8(7):e68209. doi: 10.1371/journal.pone.0068209 (PMC3699532; doi:10.1371/journal.pone.0068209)
Supplement: Table S5 — Primers used for RT-PCR and real-time PCR. (DOC) [file pone.0068209.s006.doc]

Table S5 Primers used for stem-loop RT-PCR

| MicroRNA | Primer | Primer sequence(5'-3') |
| --- | --- | --- |
| miR-N45 | RT | CTCAACTGGTGTCGTGGAGTCGGCAATTCAGTTGAGGCTTCGAC |
|  | Forward | ACACTCCAGCTGGGACGCGAGACGCGACG |
| miR-N24 | RT | CTCAACTGGTGTCGTGGAGTCGGCAATTCAGTTGAGGTAAACGT |
|  | Forward | ACACTCCAGCTGGGTTTCTCTCGGGCGT |
| miR-N58 | RT | CTCAACTGGTGTCGTGGAGTCGGCAATTCAGTTGAGCTAAATCA |
|  | Forward | ACACTCCAGCTGGGTACCGATTGAATG |
| miR-N50 | RT | CTCAACTGGTGTCGTGGAGTCGGCAATTCAGTTGAGTCTGCACGC |
|  | Forward | ACACTCCAGCTGGGCGGTGTTTCGTTCCAA |
| miR-N5 | RT | CTCAACTGGTGTCGTGGAGTCGGCAATTCAGTTGAGGGTTTAAC |
|  | Forward | ACACTCCAGCTGGGATTAGGATTTTTGTT |
| miR-N11 | RT | CTCAACTGGTGTCGTGGAGTCGGCAATTCAGTTGAGGGTCAACT |
|  | Forward | ACACTCCAGCTGGGGTTGTTGGGAAGTT |
| miR-N47* | RT | CTCAACTGGTGTCGTGGAGTCGGCAATTCAGTTGAGGGTACCCC |
|  | Forward | ACACTCCAGCTGGGATCAGCGGTGGTCT |
| miR-N47 | RT | CTCAACTGGTGTCGTGGAGTCGGCAATTCAGTTGAGAGGATCAGC |
|  | Forward | ACACTCCAGCTGGGTATTCGAGACCTCT |
| miR-N2 | RT | CTCAACTGGTGTCGTGGAGTCGGCAATTCAGTTGAGCGTCTTAAAT |
|  | Forward | ACACTCCAGCTGGGACCCGTTCGTCGTGG |
| miR-N57 | RT | CTCAACTGGTGTCGTGGAGTCGGCAATTCAGTTGAGCGAAACGT |
|  | Forward | ACACTCCAGCTGGGCGCGAAAATCGTCTT |
| U6 | Forward | CGTATACTAAAATTGGAACGATACAG |
|  | Revers | ATTTTGCGTGTCATCCTTGC |
| Universal revers | | TGGTGTCGTGGAGTCG |
