# Supplementary material for: Involvement of MicroRNAs in Infection of Silkworm with Bombyx mori Cytoplasmic Polyhedrosis Virus (BmCPV)
Source: PLoS One. 2013 Jul 2;8(7):e68209. doi: 10.1371/journal.pone.0068209 (PMC3699532; doi:10.1371/journal.pone.0068209)
Supplement: Table S6 — Differentially expressed miRNAs between BmCPV-infected 72 h midgut and normal midgut of silkworm. (DOC) [file pone.0068209.s007.doc]

Table S6 Differentially expressed miRNAs between BmCPV-infected 72h midgut and normal midgut of silkworm

| MicroRNA | P-Value | Ratio | Mark |
| --- | --- | --- | --- |
| bmo-miR-2843-2* | 0.000536 | 15.25 | Up |
| bmo-miR-3203 | 0.003177 | 11.86 | Up |
| bmo-miR-13a | 0.015289 | 1.88 | Up |
| bmo-miR-2769 | 0.000267 | 0.66 | Down |
| bmo-miR-275 | 0.000042 | 0.65 | Down |
| bmo-miR-10* | 0 | 0.64 | Down |
| bmo-miR-9c* | 0 | 0.63 | Down |
| bmo-miR-2774a | 0.00004 | 0.62 | Down |
| bmo-bantam | 0 | 0.62 | Down |
| bmo-miR-14 | 0 | 0.62 | Down |
| bmo-miR-2778b | 0.000132 | 0.62 | Down |
| bmo-miR-13b | 0.003723 | 0.62 | Down |
| bmo-miR-71* | 0 | 0.61 | Down |
| bmo-miR-279a | 0 | 0.61 | Down |
| bmo-miR-1a | 0 | 0.61 | Down |
| bmo-miR-281* | 0 | 0.60 | Down |
| bmo-miR-2778a* | 0.000118 | 0.60 | Down |
| bmo-miR-2778b* | 0.000118 | 0.60 | Down |
| bmo-miR-2778c | 0.000118 | 0.60 | Down |
| bmo-miR-2778d | 0.000118 | 0.60 | Down |
| bmo-miR-34 | 0 | 0.59 | Down |
| bmo-miR-277 | 0 | 0.59 | Down |
| bmo-miR-3219 | 0.022435 | 0.57 | Down |
| bmo-miR-993b* | 0.000266 | 0.55 | Down |
| bmo-miR-87 | 0 | 0.54 | Down |
| bmo-miR-92a | 0.010965 | 0.52 | Down |
| bmo-miR-316 | 0.000009 | 0.52 | Down |
| bmo-miR-282 | 0.000053 | 0.51 | Down |
| bmo-miR-2755* | 0.041782 | 0.50 | Down |
| bmo-miR-3001 | 0 | 0.47 | Down |
| bmo-miR-2843 | 0.000343 | 0.46 | Down |
| bmo-miR-307 | 0.00054 | 0.45 | Down |
| bmo-miR-79* | 0.040451 | 0.45 | Down |
| bmo-miR-9b* | 0.040451 | 0.45 | Down |
| bmo-miR-2756 | 0 | 0.37 | Down |
| bmo-miR-274 | 0.000004 | 0.33 | Down |
| bmo-miR-2778d* | 0.004688 | 0.33 | Down |
| bmo-miR-9a | 0 | 0.24 | Down |
| bmo-miR-9a* | 0.003953 | 0.24 | Down |
| bmo-bantam* | 0.006951 | 0.22 | Down |
| bmo-miR-2778c* | 0.008874 | 0.18 | Down |
| bmo-miR-2792-3p | 0.020282 | 0.13 | Down |
| bmo-miR-2826 | 0.001029 | 0.13 | Down |
| bmo-miR-927 | 0.014658 | 0.12 | Down |
| bmo-miR-306b | 0.010841 | 0.00 | Down |
| bmo-miR-274* | 0.042109 | 0.00 | Down |
| bmo-miR-2777 | 0.029798 | 0.00 | Down |
| miR-3351 | 0.005271 | 0.25 | Down |
| Novel-17* | 0.024151 | 10000.00 | Up |
| Novel-46* | 0.000585 | 6.78 | Up |
| Novel-46 | 0 | 6.65 | Up |
| Novel-11 | 0 | 6.10 | Up |
| Novel-33 | 0.000001 | 4.12 | Up |
| Novel-50 | 0 | 3.16 | Up |
| Novel-41 | 0.004895 | 2.49 | Up |
| Novel-54* | 0.016501 | 2.40 | Up |
| Novel-11* | 0 | 2.38 | Up |
| Novel-10 | 0 | 2.02 | Up |
| Novel-30* | 0.005226 | 1.98 | Up |
| Novel-45 | 0 | 1.87 | Up |
| Novel-51* | 0 | 1.86 | Up |
| Novel-52* | 0 | 1.83 | Up |
| Novel-45* | 0.00437 | 1.77 | Up |
| Novel-42 | 0 | 1.62 | Up |
| Novel-24* | 0 | 1.62 | Up |
| Novel-51 | 0.000043 | 1.61 | Up |
| Novel-6*-2 | 0.014628 | 1.54 | Up |
| Novel-1 | 0.040544 | 0.67 | Down |
| Novel-48 | 0.000336 | 0.65 | Down |
| Novel-3 | 0.005786 | 0.54 | Down |
| Novel-7 | 0.003261 | 0.50 | Down |
| Novel-19 | 0.044498 | 0.48 | Down |
| Novel-47* | 0 | 0.47 | Down |
| Novel-53 | 0 | 0.45 | Down |
| Novel-24 | 0 | 0.38 | Down |
| Novel-39 | 0.034948 | 0.37 | Down |
| Novel-12 | 0.040669 | 0.30 | Down |
| Novel-28 | 0.000288 | 0.18 | Down |

**footnote: fold change >= 1.5 or <= 0.667 and P value <= 0.05**
