# Supplementary material for: Involvement of MicroRNAs in Infection of Silkworm with Bombyx mori Cytoplasmic Polyhedrosis Virus (BmCPV)
Source: PLoS One. 2013 Jul 2;8(7):e68209. doi: 10.1371/journal.pone.0068209 (PMC3699532; doi:10.1371/journal.pone.0068209)
Supplement: Table S7 — Differentially expressed miRNAs between BmCPV-infected 96 h midgut and normal midgut of silkworm. (DOC) [file pone.0068209.s008.doc]

Table S7 Differentially expressed miRNAs between BmCPV-infected 96h midgut and normal midgut of silkworm

| MicroRNA | P-Value | Ratio | Mark |
| --- | --- | --- | --- |
| bmo-miR-2772b | 0.035191 | 10000 | Up |
| bmo-miR-2779 | 0 | 3.04597 | Up |
| bmo-miR-275 | 0 | 2.148937 | Up |
| bmo-miR-275* | 0.000073 | 1.691179 | Up |
| bmo-miR-305 | 0 | 1.683691 | Up |
| bmo-miR-965 | 0.000371 | 0.664446 | Down |
| bmo-miR-993b* | 0.005918 | 0.657167 | Down |
| bmo-miR-10* | 0.000002 | 0.643532 | Down |
| bmo-miR-970 | 0.000003 | 0.643419 | Down |
| bmo-miR-283 | 0 | 0.641248 | Down |
| bmo-miR-31 | 0 | 0.63951 | Down |
| bmo-miR-279d | 0 | 0.639396 | Down |
| bmo-miR-10 | 0 | 0.638607 | Down |
| bmo-miR-3340 | 0.001539 | 0.626153 | Down |
| bmo-miR-281 | 0 | 0.624444 | Down |
| bmo-miR-281* | 0 | 0.618429 | Down |
| bmo-miR-11* | 0.01513 | 0.618335 | Down |
| bmo-miR-307 | 0.023073 | 0.610737 | Down |
| bmo-miR-2843 | 0.012124 | 0.59435 | Down |
| bmo-miR-2756 | 0.000788 | 0.589789 | Down |
| bmo-miR-33* | 0.000021 | 0.588327 | Down |
| bmo-miR-1a | 0 | 0.558344 | Down |
| bmo-miR-279a | 0 | 0.557617 | Down |
| bmo-miR-2757* | 0.026544 | 0.528081 | Down |
| bmo-miR-2760* | 0.016203 | 0.462071 | Down |
| bmo-miR-9a | 0 | 0.461637 | Down |
| bmo-miR-274 | 0.00065 | 0.456172 | Down |
| bmo-miR-282 | 0.000003 | 0.441785 | Down |
| bmo-miR-2804 | 0.000011 | 0.428587 | Down |
| bmo-miR-137 | 0.044979 | 0.422465 | Down |
| bmo-bantam* | 0.029708 | 0.415864 | Down |
| bmo-miR-2778b | 0 | 0.404312 | Down |
| bmo-miR-2755* | 0.04186 | 0.398092 | Down |
| bmo-miR-3001 | 0 | 0.389112 | Down |
| bmo-miR-2780a* | 0.038124 | 0.369657 | Down |
| bmo-miR-745 | 0 | 0.358942 | Down |
| bmo-miR-745* | 0.000436 | 0.358455 | Down |
| bmo-miR-iab-4-5p | 0.000019 | 0.326673 | Down |
| bmo-miR-2807a | 0.02859 | 0.295725 | Down |
| bmo-miR-2838 | 0.001615 | 0.272378 | Down |
| bmo-miR-9a* | 0.032316 | 0.211232 | Down |
| bmo-miR-2819 | 0.043076 | 0.147863 | Down |
| bmo-miR-2843-2* | 0.043967 | 0 | Down |
| bmo-miR-3333 | 0.043967 | 0 | Down |
| bmo-miR-3391 | 0.043967 | 0 | Down |
| miR-2478 | 0 | 1.851148 | Up |
| miR-3351 | 0.042606 | 0.277242 | Down |
| Novel-31* | 0.000667 | 6.160942 | Up |
| Novel-56 | 0 | 4.230514 | Up |
| Novel-43 | 0.03814 | 3.450128 | Up |
| Novel-54* | 0.011691 | 3.252978 | Up |
| Novel-17 | 0.005126 | 3.062868 | Up |
| Novel-21 | 0.005126 | 3.062868 | Up |
| Novel-30* | 0.00094 | 3.031184 | Up |
| Novel-45 | 0 | 2.825232 | Up |
| Novel-6*-1 | 0.000003 | 2.614 | Up |
| Novel-52 | 0 | 2.592085 | Up |
| Novel-39 | 0.033003 | 2.587596 | Up |
| Novel-2 | 0 | 2.454519 | Up |
| Novel-30 | 0 | 2.235335 | Up |
| Novel-58* | 0 | 2.184712 | Up |
| Novel-58 | 0 | 2.14923 | Up |
| Novel-49 | 0 | 2.124058 | Up |
| Novel-6 | 0 | 2.087253 | Up |
| Novel-45* | 0.032443 | 2.083519 | Up |
| Novel-42* | 0.00521 | 1.982703 | Up |
| Novel-10 | 0 | 1.863685 | Up |
| Novel-51 | 0.000041 | 1.798627 | Up |
| Novel-2* | 0 | 1.667502 | Up |
| Novel-31 | 0.000037 | 1.632345 | Up |
| Novel-24* | 0.000004 | 1.603657 | Up |
| Novel-42 | 0.000661 | 1.5677 | Up |
| Novel-54 | 0.009854 | 1.552557 | Up |
| Novel-52* | 0.003944 | 1.509431 | Up |
| Novel-55 | 0 | 0.585871 | Down |
| Novel-48 | 0.000079 | 0.58529 | Down |
| Novel-14 | 0.025213 | 0.492875 | Down |
| Novel-61 | 0.00005 | 0.40453 | Down |
| Novel-28 | 0.027574 | 0.3943 | Down |
| Novel-50 | 0 | 0.378641 | Down |
| Novel-50* | 0 | 0.366234 | Down |
| Novel-24 | 0 | 0.300299 | Down |
| Novel-12 | 0 | 0.285164 | Down |
| Novel-5 | 0 | 0.267161 | Down |
| Novel-53* | 0.008558 | 0.246438 | Down |
| Novel-53 | 0 | 0.224343 | Down |

**footnote: fold change >= 1.5 or <= 0.667 and P value <= 0.05**
